# CRP in Outpatients with Inflammatory Bowel Disease Is Linked to the Blood Microbiota

**DOI:** 10.3390/ijms241310899

**Published:** 2023-06-30

**Authors:** Jie Xu, Göran Molin, Sanna Davidson, Bodil Roth, Klas Sjöberg, Åsa Håkansson

**Affiliations:** 1 Department of Food Technology, Engineering and Nutrition, Lund University, 22100 Lund, Sweden; susie_jie.xu@food.lth.se (J.X.); goran.molin@food.lth.se (G.M.); 2 Department of Clinical Sciences, Lund University, 21428 Malmö, Sweden; sanna.davidson@med.lu.se (S.D.); bodil.roth@med.lu.se (B.R.); klas.sjoberg@med.lu.se (K.S.); 3 Department of Gastroenterology and Nutrition, Skåne University Hospital, 20502 Malmö, Sweden

**Keywords:** *Achromobacter*, blood, microbiota, Crohn’s disease, IBD, ulcerative colitis, diversity, CRP

## Abstract

The circulation is a closed system that has been assumed to be free from bacteria, but evidence for the existence of a low-density blood microbiota is accumulating. The present study aimed to map the blood microbiota of outpatients with Crohn’s disease (CD) or with ulcerative colitis (UC) by 16S metagenomics. A diverse microbiota was observed in the blood samples. Regardless of the type of disease, the alpha diversity of the microbiota was positively associated with C-reactive protein (CRP). The blood microbiota had a surprisingly high proportion of *Proteobacteria* in comparison with human oral and colonic microbiotas. There was no clear difference in the overall pattern of the microbiota between CD and UC. A non-template control (NTC) was included in the whole process to control for the potential contamination from the environment and reagents. Certain bacterial taxa were concomitantly detected in both blood samples and NTC. However, *Acinetobacter*, *Lactobacillus*, *Thermicanus* and *Paracoccus* were found in blood from both CD and UC patients but not in NTC, indicating the existence of a specific blood-borne microbiota in the patients. *Achromobacter* dominated in all blood samples, but a minor amount was also found in NTC. *Micrococcaceae* was significantly enriched in CD, but it was also detected in high abundance in NTC. Whether the composition of the blood microbiota could be a marker of a particular phenotype in inflammatory bowel disease (IBD) or whether the blood microbiota could be used for diagnostic or therapeutic purposes deserves further attention.

## 1. Introduction

The circulation is a closed system, and for a long time, the blood in healthy individuals has been assumed to be free from bacteria, a presumption that is a prerequisite for safe blood transfusions. However, viable bacteria have been found in blood from healthy blood donors and individuals [1,2,3]. Consequently, the suspicions about the existence of a blood microbiota in non-septic individuals are accumulating [4]. On the other hand, the origins, identities and putative functions of these unanticipated micro-organisms are unknown and open for speculation. For example, it is not known how the blood microbiota is linked to the microbiota of other locations such as the gastro-intestinal (GI) tract or how the blood microbiota will be affected by diseases that increase bacterial translocation through the mucosa, e.g., in immune-driven diseases such as Crohn’s disease (CD). 

Inflammatory bowel diseases (IBDs) such as ulcerative colitis (UC) and CD are public health problems, but the aetiology of IBDs is obscure. Microbial agents seem to be involved in the pathogenesis, and intestinal bacteria can be one contributing factor to the development and progression of these diseases [5,6,7]. Dysbiosis is found in IBD patients, but other taxonomic alterations are revealed during periods of both clinical remission and relapse [8,9]. The human GI tract harbours bacteria in abundance, and these bacteria not only play a role in intestinal homeostasis and function but also in the onset and perpetuation of chronic intestinal inflammation. On the one hand, innate tolerance to commensal microorganisms must be maintained. On the other, antagonistic interactions between the host and the pathogenic organism are necessary for a favourable outcome of an infection. To cope with the situation, the body has evolved multifarious defensive and homeostatic mechanisms towards microbial infections versus harmless colonization [10]. In IBD, something in this complex balance has gone wrong, resulting in inflammation and an increased translocation of bacteria into circulation. Research on the IBD blood microbiota is, however, still scarce, and knowledge is lacking. In a few studies, it has been shown that the total bacterial DNA concentration is increased in peripheral blood from IBD patients compared to healthy controls irrespective of disease activity and that bacterial DNA is still detected in blood in patients in remission, although in lower concentrations than in patients with active IBD [11,12]. 

The aim of the present study was firstly, to investigate the possible existence of a blood microbiota of outpatients with CD and UC by 16S-targeted metagenomics and secondly, to compare the blood microbiota with clinical characteristics including commonly used biomarkers such as C-reactive protein (CRP) and albumin. Elevated serum CRP levels can indicate active inflammation although some patients may show normal levels. Despite the disadvantages of CRP such as low specificity and variability in individuals, it is a relatively inexpensive test that is widely adopted by clinical laboratories for the rapid assessment of inflammation and disease activity in IBD patients [13,14]. In contrast to CRP, serum albumin is used as a long-term marker of disease severity and nutritional status [15,16]. Faecal calprotectin, among others, is another biomarker that can also aid in the diagnosis of IBD [14,17]. 

The present work is a pilot study on the investigation of the putative translocation of the microbiota into blood circulation in IBD patients. 

## 2. Results

### 2.1. Patient Characteristics

In the whole group of IBD patients, the mean age was 43 years (range 19–70), including eleven women. In the CD subgroup, the mean age was 42 years (range 23–70), including seven women, and six patients had ileal-, three had colonic- and ten had ileocolonic involvement (Table 1). In the UC subgroup, the mean age was 46 years (range 19–68), with four women, and nine patients had extensive colitis, and five had left-sided colitis. The mean disease duration for the UC subgroup was 12 years (range 0–46 years), and for the CD subgroup, 14 years (0–46). Calprotectin taken during the visit was only available in five of the studied patients, and the results are therefore not reported. CRP had a skewed distribution with a median of 2.2 (IQR 1.0–6.3). At the visits, all patients that participated in the trial came for a routine check-up and not for any extra visit due to flare-ups. One patient had a CRP of 67 and therefore had verified active disease, while the remaining 32 patients had CRP levels ranging between 0.6 and 17. Five patients felt well, two did not report any symptoms at all, eleven had diarrhoea and four had diarrhoea with abdominal pain. Five reported only abdominal pain, and four had blood in their stool. Two patients had been treated with antibiotics during the last two months, but their microbiota did not differ in any substantial way from the others. 

### 2.2. Blood Microbiota in IBD Patients

**Figure 1:**
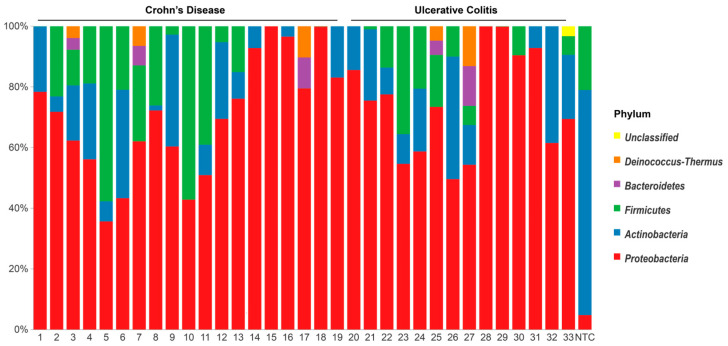
Composition of the blood microbiota detected in the IBD (*n* = 33) and NTC (*n* = 1) at the phylum level.

Analysis of the bacterial microbiota in the blood of IBD patients revealed a diverse range of bacterial DNA affiliated with different taxa in the blood samples. Contamination from the environment or reagents was detected in the NTC. The composition of the microbiota from blood samples differed from the one detected in NTC, which indicated the existence of a true blood microbiota in the studied patients (Table 2, Figure 1).

*Proteobacteria* dominated the blood samples at the phylum level, whereas *Actinobacteria* was predominant in NTC (Figure 1). The phyla detected in the CD group were *Proteobacteria*, *Actinobacteria*, *Firmicutes*, *Bacteroidetes* and a group of “unclassified bacteria”. The same phyla were found in the UC group, and in addition, *Deinococcus-Thermus* was detected in one UC sample. The NTC harboured mainly *Actinobacteria* and *Firmicutes* and only to a lesser extent *Proteobacteria*.

**Figure 2:**
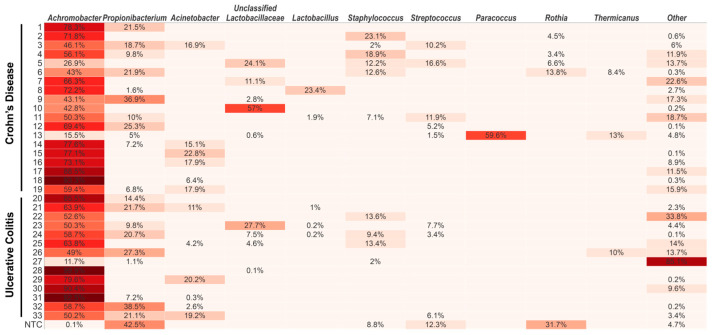
Prevalence of *Achromobacter* in blood samples of IBD patients. Top-ten abundant genera are shown. PCR-grade water served as the NTC (non-template control). Relative abundance > 0.1% is shown. The higher the value, the darker the color.

The genus *Achromobacter*, which is affiliated with the family *Alcaligenaceae* (class *Betaproteobacteria*; phylum *Proteobacteria* also named *Pseudomonadota*), dominated in abundance (median [IQR], 63.8% [50.2–77.6%]) and was found in all blood samples (Figure 2) and to a minor extent in the NTC (0.07%). The top ten abundant genera are shown in Figure 2. The taxa that are included under the heading “others” comprised 45 different genera. The detailed taxonomic identities and abundance levels are depicted in the Supplementary Materials Table S1. Abundant genera found in both blood samples and in the NTC were *Probionibacterium*, *Staphylococcus*, *Streptococcus* and *Rothia* (Figure 1). Genera found only in blood samples were *Acinetobacter* (18/33 samples), unclassified *Lactobacillaceae* (13/33) and *Lactobacillus* (10/33), *Paracoccus* (7/33) and *Thermicanus* (5/33).

**Figure 3:**
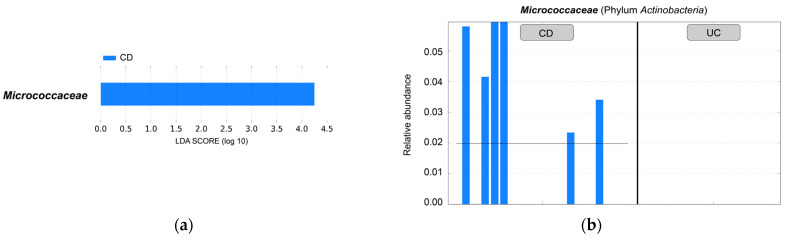
LefSe analysis. (**a**) Significantly enriched *Micrococcaceae* in the blood of CD patients compared to UC patients. LDA > 2.5 and *p* < 0.05 were considered significant; (**b**) Relative abundance of *Micrococcaceae* detected in the blood samples of IBD patients.

*Micrococcaceae* was the only taxon that with statistical significance was enriched in the CD group compared to the UC group based on LefSe analysis (Figure 3). The genus *Rothia* which belongs to the family *Micrococcaceae*, was found in abundance in the NTC (Figure 1). 

**Figure 4:**
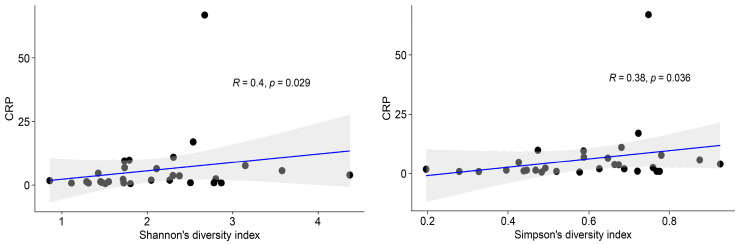
Alpha diversity, measured by Shannon’s and Simpson’s diversity indices, was positively associated with CRP.

The alpha diversity of the blood microbiota was, regardless of being CD or UC, positively associated with CRP (Figure 4). On the other hand, no statistically significant correlation was found between the alpha diversity and albumin.

Alpha diversity, measured using Shannon’s and Simpson’s diversity indices, showed no significant differences between CD and UC patients (Table 3). 

**Figure 5:**
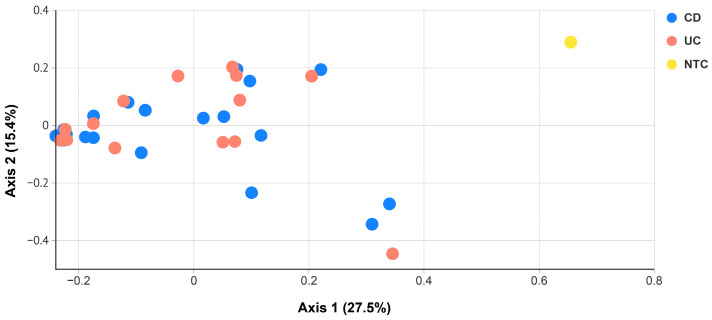
Beta diversity analysis between blood from CD (blue dots) and UC patients (red dots). ANOSIM (analysis of similarities) test using the Bray–Curtis distance matrix resulted in *p* = 0.90. NTC (yellow dot) = non-template control.

No significant differences were found between CD and UC patients in terms of beta diversity, i.e., when the microbiota of each individual blood sample was compared with each other (Figure 5). 

## 3. Discussion

*Achromobacter* was found in the blood of all patients. Even though this dominance of *Achromobacter* could be due to contamination in the clinic or in the laboratory, it may also be a consequence of colonization in the human body. Members of the genus *Achromobacter*, which belongs to the family *Alcaligenaceae* (in the class of *Gammaproteobacteria*), are ubiquitous non-fermenting, gram-negative, multi-resistant and invasive and have been linked to abscesses and deep infections [18,19]. *Achromobacter* is an opportunistic pathogen and can cause various infections in cystic fibrosis patients, as well as in hosts with different underlying conditions weakening the immune system. *Achromobacter* is predominantly recovered from patients with cystic fibrosis as a pathogen involved in chronic pulmonary colonization. Outside the context of cystic fibrosis, pneumonia and bacteremia are the two most common clinical presentations of *Achromobacter* infections [20,21]. Occasionally, *Achromobacter* infections can occur in immunocompromised individuals [22]. *Achromobacter* can penetrate the mucosa and aggravate the immunological defence, especially if the barrier effect is weakened. On the other hand, this type of microorganism can also be expected in cases of environmental contamination. A commonly reported contaminated germicide is chlorhexidine solution, used as both an antiseptic and a disinfectant. Atomizers, dispensers and various product containers were identified as reservoirs [19]. Awareness of the high survival ability of *Achromobacter* in germicides and the possible hospital reservoirs of these microbes will help to improve infection control and prevent nosocomial outbreaks or pseudo-outbreaks caused by *Achromobacter* [19]. The majority of *Achromobacter* infections are acquired nosocomial, as this bacterium can colonize various medical devices and could be found in contaminated solutions used in hospitals, such as dialysis water, demineralized water, humidifiers and even antiseptic and disinfectant solutions. Not only are *Achromobacter* species able to establish chronic infections, they can also resist common disinfectants and readily acquire antibiotic resistance [21]. *Achromobacter* species are frequently carriers of antimicrobial resistant genes and therefore contribute to the global public health threat of antibiotic resistance. Under the condition that the *Achromobacter* species in the present study primarily originated from blood and not from a contaminated environment, *Achromobacter* species seem to be more prone than many other taxa to translocate through the mucous membrane and find their way into circulation. In a mouse model, *Achromobacter* could translocate to mesenteric adipose tissue and exacerbate colitis in mice [23]. This is puzzling as *Achromobacter* are gram-negative bacteria with lipopolysaccharides (LPS) in the cell wall, and LPS are generally assumed to be strong immune-aggravating components. On the other hand, *Achromobacter* species seem to have several strategies for immune evasion. In patients with cystic fibrosis, *Achromobacter* species survive in the lungs under the selective pressure imposed by the host immune system and antibiotic therapies by increasing efficiency in nutrient acquisition, developing the ability to avoid toxic compounds and to evade the immune response and in this way, promote the colonization of new areas [24]. In a systematic review on nosocomial outbreaks of bacteraemia due to *Achromobacter*, seven studies were selected, and for true or pseudo-bacteraemia, positive blood culture results were most commonly reported in immunosuppressed patients or those with indwelling catheters [19]. An increase in *Achromobacter* species in the colon mucosa of patients during the exacerbated phase of ulcerative colitis has been observed [25]. Further investigation is needed to determine the origin of *Achromobacter* species detected in the blood samples of IBD patients in the present study.

Abundant genera found in blood in the present study, but not in the negative control, were *Acinetobacter*, unclassified *Lactobacillaceae* and *Lactobacillus*, *Paracoccus* and *Thermicanus* in both CD and UC. Even if *Achromobacter* occurred due to environmental contamination, it is less likely that the presence of these other genera were caused by contamination as well. They are not normally related to skin and chemical reagents, and the concomitant occurrence of several different genera also makes environmental contamination less likely. *Acinetobacter*, a genus of gram-negative bacteria belonging to the class *Gammaproteobacteria* (phylum: *Proteobacteria*), is just as widely spread as *Achromobacter* in the environment by soil and water and is commonly a carrier of resistance to multiple antimicrobial agents [26]. The species *Acinetobacter baumannii* has emerged as a major cause of healthcare-associated infections and, for example, *Acinetobacter guillouiae* can cause sepsis by other means [27]. *Paracoccus* species are widely spread in soil and water. These are gram-negative organisms that belong to the class *Alphaproteobacteria* [28]. At present, there is only one described species of the gram-positive genus *Thermicanus* (class: *Bacilli*; phylum *Firmicutes* also named *Bacillota*), and that is *T. aegyptius*, originally isolated from soil [29].

An important finding of the present study is the positive association between the bacterial diversity and the increased CRP (Figure 4). Increased bacterial diversity regardless of dead or live microorganisms is a direct indication of increased translocation. Although found with high prevalence, *Achromobacter* alone is not directly linked to the increased CRP. Thus, it is also interesting to look further into other taxa detected in lower abundance in the blood. The increase in CRP is probably linked to an increased number of bacteria and the presence of more pro-inflammatory taxa. Looking at the taxa that could be identified to the species level in the present study, a majority of them are well-known opportunistic pathogens (Table S2). The high proportion of well-known opportunistic pathogens is striking in Table S2; and six out of nine are *Proteobacteria*. Taking the whole blood microbiota into account, *Proteobacteria* is the dominant phylum (see the Supplementary Table S1). This is in contrast to the frequently reported microbiotas of the GI tract, which is usually dominated by *Firmicutes* and *Bacteroidetes* [30,31]. Despite being gram-positive (no LPS in the cell wall) and belonging to the phylum *Firmicutes*, the genera *Staphylococcus* and *Streptococcus* in Figure 2 include many notoriously pathogenic species and can therefore be suspected to act in a pro-inflammatory way. However, the pathogenicity varies within wide limits between different species and strains, especially within the genus *Streptococcus*. *Propionibacterium* and *Acinetobacter* are also genera that include some pathogenic or opportunistic pathogenic species and can, depending on the species, be suspected to execute pro-inflammatory effects on the immune system. *Staphylococcus* and *Propionibacterium* are typically and frequently found in high numbers on the skin [32]. *Streptococcus* is typically found in the mouth and in the small intestine, while *Rothia* can be found in various sites of the GI tract. IBD patients are also well known to harbour an unbalanced gut microbiota with an abundance of taxa with pro-inflammatory capacity, for example, taxa belonging to the family *Enterobacteriaceae* and the genus *Bacteroides* [33,34,35,36]. These bacterial groups have lipopolysaccharides (LPS) associated to the cell wall, and a translocation into circulation will certainly aggravate inflammatory markers such as CRP. 

Blood from CD patients in the present study had a higher abundance of *Micrococcaceae* than blood from UC patients. *Rothia*, *Micococcus* and *Nesterenkonia* are genera belonging to the family *Micrococcaceae*. *Rothia* was dominant, but the relative abundance of only *Rothia* was not significantly different between the two groups of patients. Primary sclerosing cholangitis (PSC) is a liver disease known for its frequent concurrence with inflammatory bowel disease. When the salivary microbial communities of PSC patients and UC patients were compared with healthy controls, the beta diversity showed significant differences among the three groups. Taxonomic assignment revealed that the PSC salivary microbiotas were characterized by significant decreases in the abundances of *Rothia* and *Haemophilus* compared to the control group [37]. The faecal microbiotas of patients with PSC and UC had low diversity. *Rothia*, *Enterococcus*, *Streptococcus* and *Veillonella* were markedly overrepresented in PSC, regardless of concomitant UC [38]. The family *Lactobacillaceae* was surprisingly detected in the blood of 17 out of 33 patients. All taxa of this family are generally regarded as safe (Table S2). 

Of course, there are some limitations in the present study. Unfortunately, the majority of the patients did not have sufficient samples for the analysis of calprotectin, which could have contributed to a better evaluation of disease activity than CRP or subjectively reported symptoms. On the other hand, the consecutive recruitment resulted in a cohort of patients that had mild to moderate symptoms that could be expected at regular visits. Patients without any complaints tend to postpone their visit to a later occasion. Furthermore, no biopsies were available, which could also have given information about disease activity. Mucosal samples for determination of the mucosal flora could also have been obtained in that case. Another problem with this type of study is the risk of contamination that must always be taken into consideration. Repeated investigations with meticulous precautions are crucial to avoid false-positive results. In the present study, we included a non-template control for controlling the laboratory process. However, we did not have controls from healthy subjects for comparison. The research on blood microbiota is scarce, and our first aim was to investigate the mere existence of any blood microbiota in the IBD patients. We acknowledge the need for and importance of including samples from healthy subjects in future studies to clarify the potential differences in blood microbiota between healthy and IBD patients. Furthermore, a comparison between microbiota in the gut (but also other sites) and the blood is warranted in order to clarify the origin of the blood microbiota. A strength is the NGS analysis that provides a full picture of both culturable and non–culturable species. Furthermore, to the best of our knowledge, this is the first report about blood microbiota in IBD and its relation to CRP as a marker of inflammatory activity. The consecutive recruitment of IBD patients attending an out-patient clinic for regular visits illustrates the varying degree of disease activity that can be seen in a real-world scenario. 

## 4. Materials and Methods

### 4.1. Study Design

Patients attending the outpatient clinic at the Department of Gastroenterology and Nutrition at Skåne University Hospital, Malmö, Sweden, for regular check-ups were consecutively recruited and invited to participate. Any antibiotic treatment during the last two months was recorded. After written informed consent, blood samples were drawn from 19 outpatients with CD and 14 outpatients with UC. Standard procedures with regard to antiseptic sampling and sterile equipment were applied. The blood samples were frozen immediately and stored at −80 °C until analysis. The study was approved by the Swedish Ethical Review Authority (protocol number 2018-577). Written informed consent was obtained from all participating patients. All methods used in this study were carried out in accordance with relevant guidelines and regulations. 

### 4.2. 16S-Targeted Metagenomics

The frozen whole blood samples were thawed, and the total DNA was extracted using the EZ1&2 DNA Tissue kit (Qiagen, Hilden, Germany) with minor modification. A bead beading step was added to aid the bacterial cell lysis prior to the automated protocol for the DNA extraction on the EZ1 Advanced XL BioRobot (Qiagen). A non-template control (NTC) was included from the DNA extraction and downstream of sequencing. To increase the specificity of the bacterial DNA amplification, a nested PCR was performed. First, PCR was performed targeting a longer region of 16S (V1–V4) with the AllTaq PCR core kit (Qiagen) using a primer pair (ENV1 5′-AGAGTTTGATIITGGCTCAG-3′ and 805R 5′-CTACHVGGGTATCTAATCC-3′). The PCR was performed at 94 °C for 3 min followed by 25 cycles at 94 °C for 1 min, 50 °C for 45 s and 72 °C for 2 min. The amplicons were cleaned with a QIAquick PCR purification kit (Qiagen). Then, the purified amplicons were used as a template for 16S V3–V4 amplicon sequencing following the Illumina 16S Metagenomic Sequencing Library Preparation protocol. A final library of 6 pM with 5% PhiX spike-in was loaded on a MiSeq sequencer using the Miseq reagent kit V3 (600 cycle). 

Sequence data analysis was performed using an open-source bioinformatics tool, i.e., Qiime2 (2022.2) [39]. Briefly, adapter and primer sequences were trimmed using the cutadapt plugin [40]. Then, the sequences were further processed with the dada2 plugin [41]. The identified amplicon sequence variants (ASVs) were used for downstream analysis. For the classification of the bacteria, the naïve Bayes classifier was trained on the V3–V4 region of reference sequences from Greengenes 13_8 (99% sequence similarity) using the QIIME2 plugin feature-classifier [42]. Alpha diversity was estimated with Shannon and Simpson’s diversity indices, and beta diversity was calculated using the Bray–Curtis distance matrix. Sequence data were submitted to SRA with BioProject ID PRJNA954608.

### 4.3. Statistics

Most statistical tests were performed in R (version 4.2.1) [43]. For non-normally distributed data, Wilcoxon’s rank sum test and Spearman’s correlation test were used. For multiple comparisons, *p*-values were corrected with the Benjamini–Hochberg method, and *p* < 0.05 was regarded as statistically significant. Linear discriminant analysis effect size (LEfSe) analysis [44] was performed to detect differentially abundant taxa between the CD and UC groups.

## 5. Conclusions

To conclude, DNA from *Acinetobacter*, *Lactobacillus* and unclassified *Lactobacillaceae*, *Thermicanus* and *Paracoccus* was found in blood from both CD and UC but not in the control, indicating the existence of a blood-borne microbiota at least in the studied patients. It is noteworthy that these patients came for a regular check-up and not because of flare-ups. It remains to be seen whether fare-ups result in higher amounts of bacteria and other species than those found under more inactive conditions. *Acinetobacter* must be regarded as the most negative from a health point of view while *Lactobacillaceae* must be regarded as safe. If these bacteria fill any yet unknown function or are apparent just as a consequence of translocation remains to be clarified. If the blood microbiota could be used for diagnostic or therapeutic purposes, clinical work-up in these patients could be facilitated. *Achromobacter* dominated all blood samples. It is not clear if this microorganism originated from contamination or from the patients. This has to be studied further.

## Figures and Tables

**Table 1:** Demographic characteristics of the two study groups.

	Whole Group	Crohn’s Disease		Ulcerative Colitis	
Patients (*n*)	33		19		14
Age, years (range)	43 (19–70)		42 (23–70)		46 (19–69)
Duration, years (range)	12 (0–46)		14 (0–46)		9 (0–29)
Gender	11 F/22 M		7 F/12 M		4 F/10 M
Localisation		L1	6	E1	0
		L2	3	E2	5
		L3	10	E3	9
Behaviour		B1	9		
		B2	6		
		B3	2		
		B2/B3	1		
		B3/B4	1		
		Missing	0		
Smoking (*n*)					
Never	16		9		7
Past	13		6		7
Current	4		4		0
Laboratory data					
CRP, mg/L (range)	6.0 (0.6–67)		3.1 (0.8–7.7)		9.7 (0.6–67)
Median (IQR)	2.2 (1.0–6.3)		2.3 (1.1–4.7)		2.0 (0.9–9.8)
Hb, g/L (range)	134 (102–163)		136 (102–163)		132 (108–153)
Albumin, g/L (range)	41 (21–49)		40 (21–49)		41 (33–47)

Description of disease location and behaviour according to the Montreal classification: Localisation: In Crohn’s disease, L1 = ileal, L2 = colonic and L3 = ileocolonic, in ulcerative colitis, E1 = rectum (excluded due to too limited inflammation), E2 = left sided and E3 = extensive colitis; Behaviour: B1 = non-structuring, non-penetrating, B2 = structuring, B3 = penetrating and B4 = perianal disease modifier.

**Table 2:** Summary of amplicon sequence variant (ASV) counts and number of taxa detected in the blood samples and non-template control (NTC).

	ASVs *	Phylum	Family	Genus	Species
CD	108,217 (49,524–245,291)	5	28	36	43
UC	120,973 (75,627–220,242)	5	23	32	42
NTC	78,209	3	6	6	7

* ASVs for CD and UC median (min–max).

**Table 3:** Alpha diversity of the blood microbiota from IBD patients.

	Shannon’s Diversity Index Median [IQR]	Simpson’s Diversity Index Median [IQR]	*p*-Value
CD patients (*n* = 19)	2.05 [1.53–2.51]	0.65 [0.47–0.72]	0.87
UC patients (*n* = 14)	2.04 [1.46–2.57]	0.61 [0.45–0.73]	0.93

## Data Availability

All data discussed are presented within the article.

## Supplementary Materials

The supporting information can be downloaded at https://www.mdpi.com/article/10.3390/ijms241310899/s1.

## References

[1] Nikkari S., McLaughlin I.J., Bi W.L., Dodge D.E., Relman D.A. (2001). Does blood of healthy subjects contain bacterial ribosomal DNA?. J. Clin. Microbiol..

[2] Moriyama K., Ando C., Tashiro K., Kuhara S., Okamura S., Nakano S., Takagi Y., Miki T., Nakashima Y., Hirakawa H. (2008). Polymerase chain reaction detection of bacterial 16S rRNA gene in human blood. Microbiol. Immunol..

[3] Damgaard C., Magnussen K., Enevold C., Nilsson M., Tolker-Nielsen T., Holmstrup P., Nielsen C.H. (2015). Viable Bacteria Associated with Red Blood Cells and Plasma in Freshly Drawn Blood Donations. PLoS ONE.

[4] Castillo D.J., Rifkin R.F., Cowan D.A., Potgieter M. (2019). The Healthy Human Blood Microbiome: Fact or Fiction?. Front. Cell. Infect. Microbiol..

[5] Alam M.T., Amos G.C.A., Murphy A.R.J., Murch S., Wellington E.M.H., Arasaradnam R.P. (2020). Microbial imbalance in inflammatory bowel disease patients at different taxonomic levels. Gut Pathog..

[6] Frank D.N., Amand A.L.S., Feldman R.A., Boedeker E.C., Harpaz N., Pace N.R. (2007). Molecular-phylogenetic characterization of microbial community imbalances in human inflammatory bowel diseases. Proc. Natl. Acad. Sci. USA.

[7] Ott S.J., Musfeldt M., Wenderoth D.F., Hampe J., Brant O., Folsch U.R., Timmins K.N., Schreiber S. (2004). Reduction in diversity of the colonic mucosa associated bacterial microflora in patients with active inflammatory bowel disease. Gut.

[8] Pisani A., Rausch P., Bang C., Ellul S., Tabone T., Cordina C.M., Zahra G., Franke A., Ellul P. (2022). Dysbiosis in the Gut Microbiota in Patients with Inflammatory Bowel Disease during Remission. Microbiol. Spectr..

[9] Serrano-Gomez G., Mayorga L., Oyarzun I., Roca J., Borruel N., Casellas F., Varela E., Pozuelo M., Machiels K., Guarner F. (2021). Dysbiosis and relapse-related microbiome in inflammatory bowel disease: A shotgun metagenomic approach. Comput. Struct. Biotechnol. J..

[10] Khan I., Bai Y.R., Zha L.J., Ullah N., Ullah H., Shah S.R.H., Sun H., Zhang C.J. (2021). Mechanism of the Gut Microbiota Colonization Resistance and Enteric Pathogen Infection. Front. Cell. Infect. Microbiol..

[11] Vrakas S., Mountzouris K.C., Michalopoulos G., Karamanolis G., Papatheodoridis G., Tzathas C., Gazouli M. (2017). Intestinal Bacteria Composition and Translocation of Bacteria in Inflammatory Bowel Disease. PLoS ONE.

[12] Gutierrez A., Frances R., Amoros A., Zapater P., Garmendia M., NDongo M., Cano R., Jover R., Such J., Perez-Mateo M. (2009). Cytokine Association with Bacterial DNA in Serum of Patients with Inflammatory Bowel Disease. Inflamm. Bowel Dis..

[13] Ishida N., Higuchi T., Miyazu T., Tamura S., Tani S., Yamade M., Iwaizumi M., Hamaya Y., Osawa S., Furuta T. (2021). C-reactive protein is superior to fecal biomarkers for evaluating colon-wide active inflammation in ulcerative colitis. Sci. Rep.-UK.

[14] Lewis J.D. (2011). The Utility of Biomarkers in the Diagnosis and Therapy of Inflammatory Bowel Disease. Gastroenterology.

[15] Shiga H., Abe I., Onodera M., Moroi R., Kuroha M., Kanazawa Y., Kakuta Y., Endo K., Kinouchi Y., Masamune A. (2020). Serum C-reactive protein and albumin are useful biomarkers for tight control management of Crohn’s disease in Japan. Sci. Rep.-UK.

[16] Khan N., Patel D., Shah Y., Trivedi C., Yang Y.X. (2017). Albumin as a prognostic marker for ulcerative colitis. World J. Gastroenterol..

[17] Vernia F., Di Ruscio M., Stefanelli G., Viscido A., Frieri G., Latella G. (2020). Is fecal calprotectin an accurate marker in the management of Crohn’s disease?. J. Gastroen Hepatol..

[18] Gomez-Cerezo J., Suarez I., Rios J.J., Pena P., de Miguel M.J.G., de Jose M., Monteagudo O., Linares P., Barbado-Cano A., Vazquez J.J. (2003). Achromobacter xylosoxidans bacteremia: A 10-year analysis of 54 cases. Eur. J. Clin. Microbiol..

[19] Yoon S.H., Kim H., Lim S.M., Kang J.M. (2022). Nosocomial outbreak of Achromobacter spp. bacteremia due to germicide contamination: A systematic review. Eur. Rev. Med. Pharmacol. Sci..

[20] Marion-Sanchez K., Pailla K., Olive C., Le Coutour X., Derancourt C. (2019). Achromobacter spp. healthcare associated infections in the French West Indies: A longitudinal study from 2006 to 2016. BMC Infect. Dis..

[21] Isler B., Kidd T.J., Stewart A.G., Harris P., Paterson D.L. (2020). Achromobacter Infections and Treatment Options. Antimicrob. Agents Chemother..

[22] Weitkamp J.H., Tang Y.W., Haas D.W., Midha N.K., Crowe J.E. (2000). Recurrent Achromobacter xylosoxidans bacteremia associated with persistent lymph node infection in a patient with hyper-immunoglobulin M syndrome. Clin. Infect. Dis..

[23] He Z., Wu J.J., Gong J.L., Ke J., Ding T., Zhao W.J., Cheng W.M., Luo Z.H., He Q.L., Zeng W.Y. (2021). Microbiota in mesenteric adipose tissue from Crohn’s disease promote colitis in mice. Microbiome.

[24] Houry A., Gohar M., Deschamps J., Tischenko E., Aymerich S., Gruss A., Briandet R. (2012). Bacterial swimmers that infiltrate and take over the biofilm matrix. Proc. Natl. Acad. Sci. USA.

[25] Walujkar S.A., Kumbhare S.V., Marathe N.P., Patangia D.V., Lawate P.S., Bharadwaj R.S., Shouche Y.S. (2018). Molecular profiling of mucosal tissue associated microbiota in patients manifesting acute exacerbations and remission stage of ulcerative colitis. World J. Microb. Biot..

[26] Munoz-Price L.S., Weinstein R.A. (2008). Acinetobacter infection. N. Engl. J. Med..

[27] Hafiz T.A., Alghamdi S.S., Mubaraki M.A., Alghamdi S.S.M., Alothaybi A., Aldawood E., Alotaibi F. (2023). A two-year retrospective study of multidrug-resistant Acinetobacter baumannii respiratory infections in critically Ill patients: Clinical and microbiological findings. J. Infect. Public. Health.

[28] van Spanning R.J., Stouthamer A.H., Baker S.C., van Verseveld H.W. (2015). Paracoccus. Bergey’s Manual of Systematics of Archaea and Bacteria.

[29] Gossner A.S., Devereux R., Ohnemuller N., Acker G., Stackebrandt E., Drake H.L. (1999). Thermicanus aegyptius gen. nov., sp nov., isolated from oxic soil, a fermentative microaerophile that grows commensally with the thermophilic acetogen Moorella thermoacetica. Appl. Environ. Microb..

[30] Eckburg P.B., Bik E.M., Bernstein C.N., Purdom E., Dethlefsen L., Sargent M., Gill S.R., Nelson K.E., Relman D.A. (2005). Diversity of the human intestinal microbial flora. Science.

[31] Zoetendal E.G., Rajilic-Stojanovic M., de Vos W.M. (2008). High-throughput diversity and functionality analysis of the gastrointestinal tract microbiota. Gut.

[32] Hannigan G.D., Grice E.A. (2013). Microbial Ecology of the Skin in the Era of Metagenomics and Molecular Microbiology. Csh Perspect. Med..

[33] Swidsinski A., Weber J., Loening-Baucke V., Hale L.P., Lochs H. (2005). Spatial organization and composition of the mucosal flora in patients with inflammatory bowel disease. J. Clin. Microbiol..

[34] Wang M., Molin G., Ahrne S., Adawi D., Jeppsson B. (2007). High proportions of proinflammatory bacteria on the colonic mucosa in a young patient with ulcerative colitis as revealed by cloning and sequencing of 16S rRNA genes. Dig. Dis. Sci..

[35] Imhann F., Vila A.V., Bonder M.J., Fu J.Y., Gevers D., Visschedijk M.C., Spekhorst L.M., Alberts R., Franke L., van Dullemen H.M. (2018). Interplay of host genetics and gut microbiota underlying the onset and clinical presentation of inflammatory bowel disease. Gut.

[36] Altomare A., Putignani L., Del Chierico F., Cocca S., Angeletti S., Ciccozzi M., Tripiciano C., Piccola B.D., Cicala M., Guarino M.P.L. (2019). Gut mucosal-associated microbiota better discloses inflammatory bowel disease differential patterns than faecal microbiota. Dig. Liver Dis..

[37] Iwasawa K., Suda W., Tsunoda T., Oikawa-Kawamoto M., Umetsu S., Takayasu L., Inui A., Fujisawa T., Morita H., Sogo T. (2018). Dysbiosis of the salivary microbiota in pediatric-onset primary sclerosing cholangitis and its potential as a biomarker. Sci. Rep..

[38] Bajer L., Kverka M., Kostovcik M., Macinga P., Dvorak J., Stehlikova Z., Brezina J., Wohl P., Spicak J., Drastich P. (2017). Distinct gut microbiota profiles in patients with primary sclerosing cholangitis and ulcerative colitis. World J. Gastroenterol..

[39] Bolyen E., Rideout J.R., Dillon M.R., Bokulich N.A., Abnet C.C., Al-Ghalith G.A., Alexander H., Alm E.J., Arumugam M., Asnicar F. (2019). Reproducible, interactive, scalable and extensible microbiome data science using QIIME 2. Nat. Biotechnol..

[40] Martin M. (2011). Cutadapt removes adapter sequences from high-throughput sequencing reads. EMBnet J..

[41] Callahan B.J., McMurdie P.J., Rosen M.J., Han A.W., Johnson A.J.A., Holmes S.P. (2016). DADA2: High-resolution sample inference from Illumina amplicon data. Nat. Methods.

[42] Bokulich N.A., Kaehler B.D., Rideout J.R., Dillon M., Bolyen E., Knight R., Huttley G.A., Caporaso J.G. (2018). Optimizing taxonomic classification of marker-gene amplicon sequences with QIIME 2 ’ s q2-feature-classifier plugin. Microbiome.

[43] R Core Team (2021). R: A Language and Environment for Statistical Computing.

[44] Segata N., Izard J., Waldron L., Gevers D., Miropolsky L., Garrett W.S., Huttenhower C. (2011). Metagenomic biomarker discovery and explanation. Genome Biol..

