# Supplementary material for: CRP in Outpatients with Inflammatory Bowel Disease Is Linked to the Blood Microbiota
Source: Int J Mol Sci. 2023 Jun 30;24(13):10899. doi: 10.3390/ijms241310899 (PMC10341653; doi:10.3390/ijms241310899)
Supplement: Supplementary file 1 [file ijms-24-10899-s001.zip › Table S2. Identified species in the blood of IBD patients.pdf]

Table S2. Identified species in the blood of IBD patients.

Opportunistic pathogen [29]	Species regarded as safe or neutral
Rothia mucilaginosa	*Anoxybacillus kestanbolensis*
Propionibacterium acnes [a)]	*Lactobacillus helveticus*
Enterococcus cecorum	*Lactobacillus iners*
Brevundimonas vesicularis	*Lactobacillus plantarum* [b)]
Methylobacterium adhaesivum	*Streptococcus infantis*
Neisseria subflava	*Sphingomonas wittichii* [c)]
Klebsiella oxytoca	*Variovorax paradoxus*
Haemophilus parainfluenzae	
Acinetobacter guillouiae	

[a)] *Cutibacterium acnes* ; [b)] *Lactiplantibacillus plantarum* ; [c)] *Rhizorhabdus wittichii*
