# Supplementary material for: CRP in Outpatients with Inflammatory Bowel Disease Is Linked to the Blood Microbiota
Source: Int J Mol Sci. 2023 Jun 30;24(13):10899. doi: 10.3390/ijms241310899 (PMC10341653; doi:10.3390/ijms241310899)
Supplement: Supplementary file 1 [file Table S1. Taxonomic identification with relative abundance data.pdf › Table S1. Taxonomic identification with relative abundance data.pdf]

Full taxonomic hierarchy	taxon at specific level	1 CD	2 CD	3 CD	4 CD	5 CD	6 CD	7 CD	8 CD	9 CD	10 CD	11 CD	12 CD	13 CD	14 CD	15 CD	16 CD	17 CD	18 CD	19 CD	22 UC	23 UC	24 UC	20 UC	25 UC	26 UC	27 UC	28 UC	29 UC	21 UC	30 UC	31 UC	32 UC	33 UC	NTC NTC
**Phylum**																																			
k__Bacteria;p__Proteobacteria	*Proteobacteria*	0.783686	0.717817	0.648117	0.56126	0.356547	0.432969	0.663223	0.722366	0.603561	0.428368	0.509187	0.694639	0.761035	0.92791	0.999807	0.965622	0.885423	0.99732	0.830806	0.77534	0.546345	0.586951	0.855677	0.770438	0.495989	0.625351	0.999492	0.99874	0.754862	0.904254	0.928143	0.615136	0.694382	0.047795
k__Bacteria;p__Actinobacteria	*Actinobacteria*	0.21569	0.050886	0.189199	0.250095	0.066437	0.357545	0	0.016483	0.368802	0	0.100234	0.252881	0.088002	0.071843	0	0.034106	0	0	0.168933	0.088495	0.097773	0.207136	0.144229	0	0.404038	0.150968	0	0	0.234999	0	0.071698	0.384864	0.211419	0.742037
k__Bacteria;p__Firmicutes	*Firmicutes*	0.000624	0.231297	0.122452	0.188645	0.577016	0.209487	0.267717	0.261151	0.027637	0.571632	0.390578	0.05248	0.150964	0.000247	0.000193	0.000272	0.000285	0.000314	0.000261	0.136164	0.355881	0.205913	9.31E-05	0.179991	0.099973	0.072677	0.000508	0.00126	0.010139	0.095746	0.000159	0	0.061407	0.210168
k__Bacteria;p__Bacteroidetes	*Bacteroidetes*	0	0	0.040232	0	0	0	0.06906	0	0	0	0	0	0	0	0	0	0.113728	0	0	0	0	0	0	0.049571	0	0.151003	0	0	0	0	0	0	0	0
k__Bacteria;p__[Thermi]	*[Thermi]*	0	0	0	0	0	0	0	0	0	0	0	0	0	0	0	0	0	0	0	0	0	0	0	0	0	0	0	0	0	0	0	0	0.032793	0
k__Bacteria;__	*Unclassified bacteria*	0	0	0	0	0	0	0	0	0	0	0	0	0	0	0	0	0.000564	0.002366	0	0	0	0	0	0	0	0	0	0	0	0	0	0	0	0
**Family**																																			
k__Bacteria;p__Proteobacteria;c__Betaproteobacteria;o__Burkholderiales;f__Alcaligenaceae	*Alcaligenaceae*	0.783686	0.717817	0.461301	0.56126	0.268929	0.430554	0.663138	0.72218	0.430961	0.42832	0.503937	0.694639	0.154841	0.77636	0.771597	0.731794	0.885387	0.933227	0.594573	0.526126	0.502744	0.586951	0.85557	0.638365	0.490218	0.144306	0.999436	0.79683	0.63969	0.904209	0.925446	0.587673	0.502559	0.000678
k__Bacteria;p__Actinobacteria;c__Actinobacteria;o__Actinomycetales;f__Propionibacteriaceae	*Propionibacteriaceae*	0.215556	0.000389	0.186906	0.097549	0.000241	0.219296	0	0.016483	0.368802	0	0.100234	0.252881	0.050332	0.071843	0	0	0	0	0.068494	0.000393	0.097773	0.207087	0.144229	0	0.34183	0.011402	0	0	0.217578	0	0.071698	0.384864	0.211419	0.424964
k__Bacteria;p__Firmicutes;c__Bacilli;o__Lactobacillales;f__Lactobacillaceae	*Lactobacillaceae*	0.000135	5.19E-05	0	0	0.240843	3.38E-05	0.111323	0.234663	0.027637	0.570266	0.019425	0	0.005805	0.000247	0	0	0	0	0	0	0.279107	0.077633	9.31E-05	0.045924	0	0	0.000508	0	0.010139	0	0	0	0	0
k__Bacteria;p__Proteobacteria;c__Gammaproteobacteria;o__Pseudomonadales;f__Moraxellaceae	*Moraxellaceae*	0	0	0.16943	0	0	0	2.52E-05	0.000129	0	0	0	0	0	0.15155	0.22821	0.179521	3.56E-05	0.064093	0.178665	0	3.04E-05	0	3.58E-05	0.04217	0	0	0	0.201909	0.109905	4.48E-05	0.002697	0.025891	0.191823	0.047117
k__Bacteria;p__Proteobacteria;c__Alphaproteobacteria;o__Rhodobacterales;f__Rhodobacteraceae	*Rhodobacteraceae*	0	0	0	0	0	0	5.88E-05	5.66E-05	0	4.85E-05	0	0	0.596622	0	0	0	0	0	0	0	0	0	7.16E-05	0	0	5.82E-05	5.64E-05	0	0	0	0	0	0	0
k__Bacteria;p__Actinobacteria;c__Actinobacteria;o__Actinomycetales;f__Micrococcaceae	*Micrococcaceae*	0.000135	0.044525	5.59E-05	0.033667	0.065714	0.138248	0	0	0	0	0	0	0.031236	0	0	0.034106	0	0	0	0	0	4.89E-05	0	0	0	0	0	0	0	0	0	0	0	0.317073
k__Bacteria;p__Firmicutes;c__Bacilli;o__Bacillales;f__Bacillaceae	*Bacillaceae*	0	0	0	0	0	0	0.156394	0	0	0	0	0	0.000143	0	0.000193	0.000272	0.000285	0.000314	0.000261	0	0	0	0	0	0	0	0	0.00126	0	0.095746	0.000159	0	0	0
k__Bacteria;p__Firmicutes;c__Bacilli;o__Bacillales;f__Staphylococcaceae	*Staphylococcaceae*	0.000355	0.231116	0.020194	0.188645	0.121813	0.125631	0	0	0	0	0.070915	0	0	0	0	0	0	0	0	0.136164	0	0.093774	0	0.134068	0	0.020371	0	0	0	0	0	0	0	0.087535
k__Bacteria;p__Firmicutes;c__Bacilli;o__Bacillales;f__[Thermicanaceae]	*[Thermicanaceae]*	0.000135	0.00013	0	0	0	0.083822	0	0	0	0	0	0	0.12985	0	0	0	0	0	0	0	0	0	0	0	0.099973	0	0	0	0	0	0	0	0	0
k__Bacteria;p__Bacteroidetes;c__Flavobacteriia;o__Flavobacteriales;f__Flavobacteriaceae	*Flavobacteriaceae*	0	0	0	0	0	0	0	0	0	0	0	0	0	0	0	0	0	0	0	0	0	0	0	0	0	0.151003	0	0	0	0	0	0	0	0
k__Bacteria;p__Actinobacteria;c__Actinobacteria;o__Actinomycetales;f__Microbacteriaceae	*Microbacteriaceae*	0	0	0	0	0	0	0	0	0	0	0	0	0	0	0	0	0	0	0	0	0	0	0	0	0	0.139566	0	0	0	0	0	0	0	0
k__Bacteria;p__Proteobacteria;c__Alphaproteobacteria;o__Sphingomonadales;f__Sphingomonadacea	*Sphingomonadaceae*	0	0	0	0	0	0	0	0	0	0	0	0	0	0	0	0	0	0	0	0	0	0	0	0.089903	0	0.181821	0	0	0.005267	0	0	0	0	0
k__Bacteria;p__Firmicutes;c__Bacilli;o__Lactobacillales;f__Streptococcaceae	*Streptococcaceae*	0	0	0.102258	0	0.166333	0	0	0	0	0	0.119114	0.05248	0.015166	0	0	0	0	0	0	0	0.076774	0.034506	0	0	0	0	0	0	0	0	0	0	0.061407	0.122633
k__Bacteria;p__Bacteroidetes;c__Flavobacteriia;o__Flavobacteriales;f__[Weeksellaceae]	*[Weeksellaceae]*	0	0	0.040232	0	0	0	0	0	0	0	0	0	0	0	0	0	0.113728	0	0	0	0	0	0	0	0	0	0	0	0	0	0	0	0	0
k__Bacteria;p__Proteobacteria;c__Betaproteobacteria;o__Neisseriales;f__Neisseriaceae	*Neisseriaceae*	0	0	0	0	0	0	0	0	0.009157	0	0	0	0	0	0	0	0	0	0	0.249214	0	0	0	0	0.00577	0	0	0	0	0	0	0.001572	0	0
k__Bacteria;p__Proteobacteria;c__Alphaproteobacteria;o__Caulobacterales;f__Caulobacteraceae	*Caulobacteraceae*	0	0	0	0	0	0	0	0	0	0	0	0	0	0	0	0	0	0	0	0	0	0	0	0	0	0.101189	0	0	0	0	0	0	0	0
k__Bacteria;p__Bacteroidetes;c__Cytophagia;o__Cytophagales;f__Cytophagaceae	*Cytophagaceae*	0	0	0	0	0	0	0.06906	0	0	0	0	0	0	0	0	0	0	0	0	0	0	0	0	0	0	0	0	0	0	0	0	0	0	0
k__Bacteria;p__Proteobacteria;c__Betaproteobacteria;o__Burkholderiales;f__Comamonadaceae	*Comamonadaceae*	0	0	0	0	0	0.002414	0	0	0	0	0	0	0	0	0	0.018854	0	0	0	0	0	0	0	0	0	0.096391	0	0	0	0	0	0	0	0
k__Bacteria;p__Proteobacteria;c__Alphaproteobacteria;o__Rhizobiales;f__Beijerinckiaceae	*Beijerinckiaceae*	0	0	0	0	0	0	0	0	0.163443	0	0	0	0	0	0	0	0	0	0	0	0	0	0	0	0	0	0	0	0	0	0	0	0	0
k__Bacteria;p__Actinobacteria;c__Actinobacteria;o__Actinomycetales;f__Frankiaceae	*Frankiaceae*	0	0	0	0	0	0	0	0	0	0	0	0	0	0	0	0	0	0	0.100439	0	0	0	0	0	0	0	0	0	0	0	0	0	0	0
k__Bacteria;p__Actinobacteria;c__Actinobacteria;o__Actinomycetales;f__Actinomycetaceae	*Actinomycetaceae*	0	0	0.002238	0	0.000482	0	0	0	0	0	0	0	0	0	0	0	0	0	0	0.088102	0	0	0	0	0.062208	0	0	0	0.017422	0	0	0	0	0
k__Bacteria;p__Bacteroidetes;c__Sphingobacteriia;o__Sphingobacteriales;f__Sphingobacteriaceae	*Sphingobacteriaceae*	0	0	0	0	0	0	0	0	0	0	0	0	0	0	0	0	0	0	0	0	0	0	0	0.049571	0	0	0	0	0	0	0	0	0	0
k__Bacteria;p__Firmicutes;c__Bacilli;o__Lactobacillales;f__Enterococcaceae	*Enterococcaceae*	0	0	0	0	0	0	0	0	0	0	0.181124	0	0	0	0	0	0	0	0	0	0	0	0	0	0	0	0	0	0	0	0	0	0	0
k__Bacteria;p__Proteobacteria;c__Alphaproteobacteria;o__Rhizobiales;f__Methylobacteriaceae	*Methylobacteriaceae*	0	0	0	0	0	0	0	0	0	0	0	0	0	0	0	0	0	0	0	0	0	0	0	0	0	0.089123	0	0	0	0	0	0	0	0
k__Bacteria;p__Proteobacteria;c__Alphaproteobacteria;o__Rhizobiales;f__Bradyrhizobiaceae	*Bradyrhizobiaceae*	0	0	0	0	0	0	0	0	0	0	0	0	0	0	0	0	0	0	0.057568	0	0	0	0	0	0	0	0	0	0	0	0	0	0	0
k__Bacteria;p__Actinobacteria;c__Actinobacteria;o__Actinomycetales;f__Corynebacteriaceae	*Corynebacteriaceae*	0	0.005971	0	0.118879	0	0	0	0	0	0	0	0	0	0	0	0	0	0	0	0	0	0	0	0	0	0	0	0	0	0	0	0	0	0
k__Bacteria;p__Proteobacteria;c__Gammaproteobacteria;o__Pasteurellales;f__Pasteurellaceae	*Pasteurellaceae*	0	0	0.007283	0	0.087619	0	0	0	0	0	0.00525	0	0	0	0	0	0	0	0	0	0	0	0	0	0	0	0	0	0	0	0	0	0	0
k__Bacteria;p__Firmicutes;c__Bacilli;o__Bacillales;f__Paenibacillaceae	*Paenibacillaceae*	0	0	0	0	0	0	0	0	0	0	0	0	0	0	0	0	0	0	0	0	0	0	0	0	0	0.052307	0	0	0	0	0	0	0	0
k__Bacteria;p__Firmicutes;c__Bacilli;o__Bacillales;f__[Exiguobacteraceae]	*[Exiguobacteraceae]*	0	0	0	0	0	0	0	0.026488	0	0	0	0	0	0	0	0	0	0	0	0	0	0	0	0	0	0	0	0	0	0	0	0	0	0
k__Bacteria;p__Firmicutes;c__Bacilli;o__Gemellales;f__Gemellaceae	*Gemellaceae*	0	0	0	0	0.048026	0	0	0	0	0	0	0	0	0	0	0	0	0	0	0	0	0	0	0	0	0	0	0	0	0	0	0	0	0
k__Bacteria;p__Proteobacteria;c__Gammaproteobacteria;o__Enterobacteriales;f__Enterobacteriaceae	*Enterobacteriaceae*	0	0	0.010103	0	0	0	0	0	0	0	0	0	0	0	0	0.035453	0	0	0	0	0.043571	0	0	0	0	0	0	0	0	0	0	0	0	0
k__Bacteria;p__Proteobacteria;c__Gammaproteobacteria;o__Pseudomonadales;f__Pseudomonadaceae	*Pseudomonadaceae*	0	0	0	0	0	0	0	0	0	0	0	0	0.009572	0	0	0	0	0	0	0	0	0	0	0	0	0	0	0	0	0	0	0	0	0
k__Bacteria;p__[Thermi];c__Deinococci;o__Thermales;f__Thermaceae	*Thermaceae*	0	0	0	0	0	0	0	0	0	0	0	0	0	0	0	0	0	0	0	0	0	0	0	0	0	0	0	0	0	0	0	0	0.032793	0
k__Bacteria;p__Proteobacteria;c__Alphaproteobacteria;o__Rhodospirillales;f__Acetobacteraceae	*Acetobacteraceae*	0	0	0	0	0	0	0	0	0	0	0	0	0	0	0	0	0	0	0	0	0	0	0	0	0	0.012462	0	0	0	0	0	0	0	0
k__Bacteria;p__Actinobacteria;c__Acidimicrobiia;o__Acidimicrobiales;f__C111	*C111*	0	0	0	0	0	0	0	0	0	0	0	0	0.006433	0	0	0	0	0	0	0	0	0	0	0	0	0	0	0	0	0	0	0	0	0
k__Bacteria;__;__;__;__	*Unclassified Bacteria*	0	0	0	0	0	0	0	0	0	0	0	0	0	0	0	0	0.000564	0.002366	0	0	0	0	0	0	0	0	0	0	0	0	0	0	0	0
k__Bacteria;p__Firmicutes;c__Bacilli;o__Bacillales;f__Thermoactinomycetaceae	*Thermoactinomycetaceae*	0	0	0	0	0	0	0	0	0	0.001366	0	0	0	0	0	0	0	0	0	0	0	0	0	0	0	0	0	0	0	0	0	0	0	0
**Genus**																																			
Top 10 abundant																																			
k__Bacteria;p__Proteobacteria;c__Betaproteobacteria;o__Burkholderiales;f__Alcaligenaceae;g__Achromobacte	*Achromobacter*	0.783686	0.717817	0.461301	0.56126	0.268929	0.430554	0.663138	0.72218	0.430961	0.42832	0.503937	0.694639	0.154841	0.77636	0.771597	0.731794	0.885387	0.933227	0.594573	0.526126	0.502744	0.586951	0.85557	0.638365	0.490218	0.117338	0.999436	0.79683	0.63969	0.904209	0.925446	0.587673	0.502559	0.000678
k__Bacteria;p__Actinobacteria;c__Actinobacteria;o__Actinomycetales;f__Propionibacteriaceae;g__Propionibacterium	*Propionibacterium*	0.215556	0.000389	0.186906	0.097549	0.000241	0.219296	0	0.016483	0.368802	0	0.100234	0.252881	0.050332	0.071843	0	0	0	0	0.068494	0.000393	0.097773	0.207087	0.144229	0	0.273431	0.011402	0	0	0.217578	0	0.071698	0.384864	0.211419	0.424964
k__Bacteria;p__Proteobacteria;c__Gammaproteobacteria;o__Pseudomonadales;f__Moraxellaceae;g__Acinetobacte	*Acinetobacter*	0	0	0.16943	0	0	0	2.52E-05	0.000129	0	0	0	0	0	0.15155	0.22821	0.179521	3.56E-05	0.064093	0.178665	0	3.04E-05	0	3.58E-05	0.04217	0	0	0	0.201909	0.109905	4.48E-05	0.002697	0.025891	0.191823	0
k__Bacteria;p__Firmicutes;c__Bacilli;o__Lactobacillales;f__Lactobacillaceae;__	*Unclassified Lactobacillaceae*	0.00011	0	0	0	0.240843	0	0.111302	0.00017	0.027637	0.570266	0	0	0.005805	0.000247	0	0	0	0	0	0	0.277383	0.07553	6.45E-05	0.045924	0	0	0.000508	0	0	0	0	0	0	0
k__Bacteria;p__Firmicutes;c__Bacilli;o__Lactobacillales;f__Lactobacillaceae;g__Lactobacillus	*Lactobacillus*	2.45E-05	5.19E-05	0	0	0	3.38E-05	2.1E-05	0.234493	0	0	0.019425	0	0	0	0	0	0	0	0	0	0.001725	0.002103	2.87E-05	0	0	0	0	0	0.010139	0	0	0	0	0
k__Bacteria;p__Firmicutes;c__Bacilli;o__Bacillales;f__Staphylococcaceae;g__Staphylococcus	*Staphylococcus*	0.000355	0.231116	0.020194	0.188645	0.121813	0.125631	0	0	0	0	0.070915	0	0	0	0	0	0	0	0	0.136164	0	0.093774	0	0.134068	0	0.020371	0	0	0	0	0	0	0	0.087535
k__Bacteria;p__Firmicutes;c__Bacilli;o__Lactobacillales;f__Streptococcaceae;g__Streptococcus	*Streptococcus*	0	0	0.102258	0	0.166333	0	0	0	0	0	0.119114	0.05248	0.015166	0	0	0	0	0	0	0	0.076774	0.034506	0	0	0	0	0	0	0	0	0	0	0.061407	0.122633
k__Bacteria;p__Proteobacteria;c__Alphaproteobacteria;o__Rhodobacterales;f__Rhodobacteraceae;g__Paracoccu	*Paracoccus*	0	0	0	0	0	0	5.88E-05	5.66E-05	0	4.85E-05	0	0	0.596622	0	0	0	0	0	0	0	0	0	7.16E-05	0	0	5.82E-05	5.64E-05	0	0	0	0	0	0	0
k__Bacteria;p__Actinobacteria;c__Actinobacteria;o__Actinomycetales;f__Micrococcaceae;g__Rothia	*Rothia*	0.000135	0.044525	5.59E-05	0.033667	0.065714	0.138248	0	0	0	0	0	0	0	0	0	0	0	0	0	0	0	4.89E-05	0	0	0	0	0	0	0	0	0	0	0	0.317073
k__Bacteria;p__Firmicutes;c__Bacilli;o__Bacillales;f__[Thermicanaceae];g__Thermicanus	*Thermicanus*	0.000135	0.00013	0	0	0	0.083822	0	0	0	0	0	0	0.12985	0	0	0	0	0	0	0	0	0	0	0	0.099973	0	0	0	0	0	0	0	0	0
Others																																			
k__Bacteria;p__Firmicutes;c__Bacilli;o__Bacillales;f__Bacillaceae;g__Bacillus	*Bacillus*	0	0	0	0	0	0	0.156394	0	0	0	0	0	0.000143	0	0.000193	0.000272	0.000285	0.000314	0.000261	0	0	0	0	0	0	0	0	0.00126	0	0	0.000159	0	0	0
k__Bacteria;p__Bacteroidetes;c__Flavobacteriia;o__Flavobacteriales;f__Flavobacteriaceae;g__Flavobacterium	*Flavobacterium*	0	0	0	0	0	0	0	0	0	0	0	0	0	0	0	0	0	0	0	0	0	0	0	0	0	0.151003	0	0	0	0	0	0	0	0
k__Bacteria;p__Actinobacteria;c__Actinobacteria;o__Actinomycetales;f__Microbacteriaceae;g__Microbacterium	*Microbacterium*	0	0	0	0	0	0	0	0	0	0	0	0	0	0	0	0	0	0	0	0	0	0	0	0	0	0.139566	0	0	0	0	0	0	0	0
k__Bacteria;p__Proteobacteria;c__Alphaproteobacteria;o__Sphingomonadales;f__Sphingomonadaceae;g__Sphingomona	*Sphingomonas*	0	0	0	0	0	0	0	0	0	0	0	0	0	0	0	0	0	0	0	0	0	0	0	0.089903	0	0.170221	0	0	0.005267	0	0	0	0	0
k__Bacteria;p__Bacteroidetes;c__Flavobacteriia;o__Flavobacteriales;f__[Weeksellaceae];g__Cloacibacterium	*Cloacibacterium*	0	0	0	0	0	0	0	0	0	0	0	0	0	0	0	0	0.113728	0	0	0	0	0	0	0	0	0	0	0	0	0	0	0	0	0
k__Bacteria;p__Proteobacteria;c__Betaproteobacteria;o__Neisseriales;f__Neisseriaceae;g__Neisseria	*Neisseria*	0	0	0	0	0	0	0	0	0.009157	0	0	0	0	0	0	0	0	0	0	0.249214	0	0	0	0	0.00577	0	0	0	0	0	0	0.001572	0	0
k__Bacteria;p__Proteobacteria;c__Alphaproteobacteria;o__Caulobacterales;f__Caulobacteraceae;g__Brevundimona	*Brevundimonas*	0	0	0	0	0	0	0	0	0	0	0	0	0	0	0	0	0	0	0	0	0	0	0	0	0	0.101189	0	0	0	0	0	0	0	0
k__Bacteria;p__Bacteroidetes;c__Cytophagia;o__Cytophagales;f__Cytophagaceae;g__Hymenobacte	*Hymenobacter*	0	0	0	0	0	0	0.06906	0	0	0	0	0	0	0	0	0	0	0	0	0	0	0	0	0	0	0	0	0	0	0	0	0	0	0
k__Bacteria;p__Proteobacteria;c__Betaproteobacteria;o__Burkholderiales;f__Comamonadaceae;g__Variovora	*Variovorax*	0	0	0	0	0	0	0	0	0	0	0	0	0	0	0	0	0	0	0	0	0	0	0	0	0	0.096391	0	0	0	0	0	0	0	0
k__Bacteria;p__Firmicutes;c__Bacilli;o__Bacillales;f__Bacillaceae;g__Anoxybacillus	*Anoxybacillus*	0	0	0	0	0	0	0	0	0	0	0	0	0	0	0	0	0	0	0	0	0	0	0	0	0	0	0	0	0	0.095746	0	0	0	0
k__Bacteria;p__Proteobacteria;c__Alphaproteobacteria;o__Rhizobiales;f__Beijerinckiaceae	*Unclassified Beijerinckiaceae*	0	0	0	0	0	0	0	0	0.163443	0	0	0	0	0	0	0	0	0	0	0	0	0	0	0	0	0	0	0	0	0	0	0	0	0
k__Bacteria;p__Actinobacteria;c__Actinobacteria;o__Actinomycetales;f__Frankiaceae	*Unclassified Frankiaceae*	0	0	0	0	0	0	0	0	0	0	0	0	0	0	0	0	0	0	0.100439	0	0	0	0	0	0	0	0	0	0	0	0	0	0	0
k__Bacteria;p__Actinobacteria;c__Actinobacteria;o__Actinomycetales;f__Propionibacteriaceae	*Unclassified Propionibacteriaceae*	0	0	0	0	0	0	0	0	0	0	0	0	0	0	0	0	0	0	0	0	0	0	0	0	0.068399	0	0	0	0	0	0	0	0	0
k__Bacteria;p__Actinobacteria;c__Actinobacteria;o__Actinomycetales;f__Actinomycetaceae;g__N09	*N09*	0	0	0	0	0	0	0	0	0	0	0	0	0	0	0	0	0	0	0	0	0	0	0	0	0.062208	0	0	0	0	0	0	0	0	0
k__Bacteria;p__Bacteroidetes;c__Sphingobacteriia;o__Sphingobacteriales;f__Sphingobacteriaceae	*Unclassified Sphingobacteriaceae*	0	0	0	0	0	0	0	0	0	0	0	0	0	0	0	0	0	0	0	0	0	0	0	0.049571	0	0	0	0	0	0	0	0	0	0
k__Bacteria;p__Firmicutes;c__Bacilli;o__Lactobacillales;f__Enterococcaceae;g__Enterococcus	*Enterococcus*	0	0	0	0	0	0	0	0	0	0	0.181124	0	0	0	0	0	0	0	0	0	0	0	0	0	0	0	0	0	0	0	0	0	0	0
k__Bacteria;p__Proteobacteria;c__Alphaproteobacteria;o__Rhizobiales;f__Methylobacteriaceae	*Unclassified Methylobacteriaceae*	0	0	0	0	0	0	0	0	0	0	0	0	0	0	0	0	0	0	0	0	0	0	0	0	0	0.052965	0	0	0	0	0	0	0	0
k__Bacteria;p__Proteobacteria;c__Alphaproteobacteria;o__Rhizobiales;f__Bradyrhizobiaceae;g__Bradyrhizobium	*Bradyrhizobium*	0	0	0	0	0	0	0	0	0	0	0	0	0	0	0	0	0	0	0.057568	0	0	0	0	0	0	0	0	0	0	0	0	0	0	0
k__Bacteria;p__Actinobacteria;c__Actinobacteria;o__Actinomycetales;f__Actinomycetaceae;g__Actinomyce	*Actinomyces*	0	0	0.002238	0	0.000482	0	0	0	0	0	0	0	0	0	0	0	0	0	0	0.088102	0	0	0	0	0	0	0	0	0.017422	0	0	0	0	0
k__Bacteria;p__Proteobacteria;c__Alphaproteobacteria;o__Rhizobiales;f__Methylobacteriaceae;g__Methylobacterium	*Methylobacterium*	0	0	0	0	0	0	0	0	0	0	0	0	0	0	0	0	0	0	0	0	0	0	0	0	0	0.036158	0	0	0	0	0	0	0	0
k__Bacteria;p__Actinobacteria;c__Actinobacteria;o__Actinomycetales;f__Corynebacteriaceae;g__Corynebacterium	*Corynebacterium*	0	0.005971	0	0.118879	0	0	0	0	0	0	0	0	0	0	0	0	0	0	0	0	0	0	0	0	0	0	0	0	0	0	0	0	0	0
k__Bacteria;p__Proteobacteria;c__Betaproteobacteria;o__Burkholderiales;f__Alcaligenaceae;g__Pigmentiphag	*Pigmentiphaga*	0	0	0	0	0	0	0	0	0	0	0	0	0	0	0	0	0	0	0	0	0	0	0	0	0	0.026969	0	0	0	0	0	0	0	0
k__Bacteria;p__Proteobacteria;c__Gammaproteobacteria;o__Pasteurellales;f__Pasteurellaceae;g__Haemophilu	*Haemophilus*	0	0	0.007283	0	0.087619	0	0	0	0	0	0.00525	0	0	0	0	0	0	0	0	0	0	0	0	0	0	0	0	0	0	0	0	0	0	0
k__Bacteria;p__Actinobacteria;c__Actinobacteria;o__Actinomycetales;f__Micrococcaceae;g__Micrococcus	*Micrococcus*	0	0	0	0	0	0	0	0	0	0	0	0	0.031236	0	0	0	0	0	0	0	0	0	0	0	0	0	0	0	0	0	0	0	0	0
k__Bacteria;p__Firmicutes;c__Bacilli;o__Bacillales;f__Paenibacillaceae;g__Paenibacillus	*Paenibacillus*	0	0	0	0	0	0	0	0	0	0	0	0	0	0	0	0	0	0	0	0	0	0	0	0	0	0.022386	0	0	0	0	0	0	0	0
k__Bacteria;p__Firmicutes;c__Bacilli;o__Bacillales;f__[Exiguobacteraceae];g__Exiguobacterium	*Exiguobacterium*	0	0	0	0	0	0	0	0.025534	0	0	0	0	0	0	0	0	0	0	0	0	0	0	0	0	0	0	0	0	0	0	0	0	0	0
k__Bacteria;p__Firmicutes;c__Bacilli;o__Gemellales;f__Gemellaceae;__	*Unclassified Gemellaceae*	0	0	0	0	0.046219	0	0	0	0	0	0	0	0	0	0	0	0	0	0	0	0	0	0	0	0	0	0	0	0	0	0	0	0	0
k__Bacteria;p__Proteobacteria;c__Gammaproteobacteria;o__Enterobacteriales;f__Enterobacteriaceae;g__Enterobacte	*Enterobacter*	0	0	0	0	0	0	0	0	0	0	0	0	0	0	0	0.035453	0	0	0	0	0	0	0	0	0	0	0	0	0	0	0	0	0	0
k__Bacteria;p__Actinobacteria;c__Actinobacteria;o__Actinomycetales;f__Micrococcaceae;g__Nesterenkonia	*Nesterenkonia*	0	0	0	0	0	0	0	0	0	0	0	0	0	0	0	0.034106	0	0	0	0	0	0	0	0	0	0	0	0	0	0	0	0	0	0
k__Bacteria;p__Proteobacteria;c__Gammaproteobacteria;o__Pseudomonadales;f__Pseudomonadaceae;g__Pseudomona	*Pseudomonas*	0	0	0	0	0	0	0	0	0	0	0	0	0.009572	0	0	0	0	0	0	0	0	0	0	0	0	0	0	0	0	0	0	0	0	0
k__Bacteria;p__[Thermi];c__Deinococci;o__Thermales;f__Thermaceae;g__Meiothermu	*Meiothermus*	0	0	0	0	0	0	0	0	0	0	0	0	0	0	0	0	0	0	0	0	0	0	0	0	0	0	0	0	0	0	0	0	0.032793	0
k__Bacteria;p__Proteobacteria;c__Alphaproteobacteria;o__Rhodospirillales;f__Acetobacteraceae;g__Roseomona	*Roseomonas*	0	0	0	0	0	0	0	0	0	0	0	0	0	0	0	0	0	0	0	0	0	0	0	0	0	0.012462	0	0	0	0	0	0	0	0
k__Bacteria;p__Proteobacteria;c__Gammaproteobacteria;o__Enterobacteriales;f__Enterobacteriaceae;g__Klebsiell	*Klebsiella*	0	0	0	0	0	0	0	0	0	0	0	0	0	0	0	0	0	0	0	0	0.021943	0	0	0	0	0	0	0	0	0	0	0	0	0
k__Bacteria;p__Proteobacteria;c__Alphaproteobacteria;o__Sphingomonadales;f__Sphingomonadaceae;g__Novosphingobiur	*Novosphingobium*	0	0	0	0	0	0	0	0	0	0	0	0	0	0	0	0	0	0	0	0	0	0	0	0	0	0.0116	0	0	0	0	0	0	0	0
k__Bacteria;p__Bacteroidetes;c__Flavobacteriia;o__Flavobacteriales;f__[Weeksellaceae];g__Chryseobacterium	*Chryseobacterium*	0	0	0.040232	0	0	0	0	0	0	0	0	0	0	0	0	0	0	0	0	0	0	0	0	0	0	0	0	0	0	0	0	0	0	0
k__Bacteria;p__Firmicutes;c__Bacilli;o__Bacillales;f__Paenibacillaceae;g__Brevibacillus	*Brevibacillus*	0	0	0	0	0	0	0	0	0	0	0	0	0	0	0	0	0	0	0	0	0	0	0	0	0	0.029921	0	0	0	0	0	0	0	0
k__Bacteria;p__Proteobacteria;c__Gammaproteobacteria;o__Enterobacteriales;f__Enterobacteriaceae;__	*Unclassified Enterobacteriaceae*	0	0	0.010103	0	0	0	0	0	0	0	0	0	0	0	0	0	0	0	0	0	0.021628	0	0	0	0	0	0	0	0	0	0	0	0	0
k__Bacteria;p__Actinobacteria;c__Acidimicrobiia;o__Acidimicrobiales;f__C111	*Unclassified C111*	0	0	0	0	0	0	0	0	0	0	0	0	0.006433	0	0	0	0	0	0	0	0	0	0	0	0	0	0	0	0	0	0	0	0	0
k__Bacteria;p__Proteobacteria;c__Betaproteobacteria;o__Burkholderiales;f__Comamonadaceae;g__Delftia	*Delftia*	0	0	0	0	0	0	0	0	0	0	0	0	0	0	0	0.018854	0	0	0	0	0	0	0	0	0	0	0	0	0	0	0	0	0	0
k__Bacteria;p__Proteobacteria;c__Betaproteobacteria;o__Burkholderiales;f__Comamonadaceae;g__Pelomona	*Pelomonas*	0	0	0	0	0	0.002414	0	0	0	0	0	0	0	0	0	0	0	0	0	0	0	0	0	0	0	0	0	0	0	0	0	0	0	0
k__Bacteria;__;__;__;__;__	*Unclassified Bacteria*	0	0	0	0	0	0	0	0	0	0	0	0	0	0	0	0	0.000564	0.002366	0	0	0	0	0	0	0	0	0	0	0	0	0	0	0	0
k__Bacteria;p__Firmicutes;c__Bacilli;o__Bacillales;f__Thermoactinomycetaceae	*Unclassified Thermoactinomycetaceae*	0	0	0	0	0	0	0	0	0	0.001366	0	0	0	0	0	0	0	0	0	0	0	0	0	0	0	0	0	0	0	0	0	0	0	0
k__Bacteria;p__Firmicutes;c__Bacilli;o__Gemellales;f__Gemellaceae	*Unclassified Gemellaceae*	0	0	0	0	0.001807	0	0	0	0	0	0	0	0	0	0	0	0	0	0	0	0	0	0	0	0	0	0	0	0	0	0	0	0	0
k__Bacteria;p__Firmicutes;c__Bacilli;o__Bacillales;f__[Exiguobacteraceae];__	*Unclassified Exiguobacteraceae]*	0	0	0	0	0	0	0	0.000954	0	0	0	0	0	0	0	0	0	0	0	0	0	0	0	0	0	0	0	0	0	0	0	0	0	0
k__Bacteria;p__Proteobacteria;c__Gammaproteobacteria;o__Pseudomonadales;f__Moraxellaceae;g__Enhydrobacte	*Enhydrobacter*	0	0	0	0	0	0	0	0	0	0	0	0	0	0	0	0	0	0	0	0	0	0	0	0	0	0	0	0	0	0	0	0	0	0.047117
**Species**																																			
k__Bacteria;p__Proteobacteria;c__Betaproteobacteria;o__Burkholderiales;f__Alcaligenaceae;g__Achromobacter	*Unclassified Achromobacter*	0.783686	0.717817	0.461301	0.56126	0.268929	0.430554	0.663138	0.72218	0.430961	0.42832	0.503937	0.694639	0.154841	0.77636	0.771597	0.731794	0.885387	0.933227	0.594573	0.526126	0.502744	0.586951	0.85557	0.638365	0.490218	0.117338	0.999436	0.79683	0.63969	0.904209	0.925446	0.587673	0.502559	0.000678
k__Bacteria;p__Actinobacteria;c__Actinobacteria;o__Actinomycetales;f__Propionibacteriaceae;g__Propionibacterium;s__acne	*Propionibacterium acnes*	0.215556	0.000389	0.186906	0.097549	0.000241	0.219296	0	0.016483	0.368802	0	0.100234	0.252881	0.050332	0.071843	0	0	0	0	0.068494	0.000393	0.097773	0.207087	0.144229	0	0.273431	0.011402	0	0	0.217578	0	0.071698	0.384864	0.211419	0.424964
k__Bacteria;p__Firmicutes;c__Bacilli;o__Lactobacillales;f__Lactobacillaceae;__;__	*Unclassified Lactobacillaceae*	0.00011	0	0	0	0.240843	0	0.111302	0.00017	0.027637	0.570266	0	0	0.005805	0.000247	0	0	0	0	0	0	0.277383	0.07553	6.45E-05	0.045924	0	0	0.000508	0	0	0	0	0	0	0
k__Bacteria;p__Proteobacteria;c__Gammaproteobacteria;o__Pseudomonadales;f__Moraxellaceae;g__Acinetobacter;s__guillouia	*Acinetobacter guillouiae*	0	0	0.16943	0	0	0	2.52E-05	0.000129	0	0	0	0	0	0.15155	0.22821	0.179521	3.56E-05	0.064093	0.178665	0	3.04E-05	0	3.58E-05	0.04217	0	0	0	0.201909	0.109905	4.48E-05	0.002697	0	0.191823	0
k__Bacteria;p__Proteobacteria;c__Alphaproteobacteria;o__Rhodobacterales;f__Rhodobacteraceae;g__Paracoccus	*Unclassified Paracoccus*	0	0	0	0	0	0	5.88E-05	5.66E-05	0	4.85E-05	0	0	0.444721	0	0	0	0	0	0	0	0	0	7.16E-05	0	0	5.82E-05	5.64E-05	0	0	0	0	0	0	0
k__Bacteria;p__Actinobacteria;c__Actinobacteria;o__Actinomycetales;f__Micrococcaceae;g__Rothia;s__mucilaginosa	*Rothia mucilaginosa*	0.000135	0.044525	5.59E-05	0.033667	0.065714	0.138248	0	0	0	0	0	0	0	0	0	0	0	0	0	0	0	4.89E-05	0	0	0	0	0	0	0	0	0	0	0	0.317073
k__Bacteria;p__Firmicutes;c__Bacilli;o__Bacillales;f__Bacillaceae;g__Bacillus;	*Unclassified Bacillus*	0	0	0	0	0	0	0.156394	0	0	0	0	0	0	0	0	0	0	0	0	0	0	0	0	0	0	0	0	0	0	0	0	0	0	0
k__Bacteria;p__Firmicutes;c__Bacilli;o__Bacillales;f__Staphylococcaceae;g__Staphylococcus;__	*Unclassified Staphylococcus*	0	0.231116	0.020194	0.188645	0.121813	0	0	0	0	0	0.070915	0	0	0	0	0	0	0	0	0.136164	0	0.093774	0	0	0	0.020371	0	0	0	0	0	0	0	0.087535
k__Bacteria;p__Firmicutes;c__Bacilli;o__Bacillales;f__Staphylococcaceae;g__Staphylococcus	*Unclassified Staphylococcus*	0.000355	0	0	0	0	0.125631	0	0	0	0	0	0	0	0	0	0	0	0	0	0	0	0	0	0.134068	0	0	0	0	0	0	0	0	0	0
k__Bacteria;p__Proteobacteria;c__Alphaproteobacteria;o__Rhodobacterales;f__Rhodobacteraceae;g__Paracoccus;_	*Unclassified Paracoccus*	0	0	0	0	0	0	0	0	0	0	0	0	0.151901	0	0	0	0	0	0	0	0	0	0	0	0	0	0	0	0	0	0	0	0	0
k__Bacteria;p__Firmicutes;c__Bacilli;o__Bacillales;f__[Thermicanaceae];g__Thermicanus	*Unclassified Thermicanus*	0.000135	0.00013	0	0	0	0.083822	0	0	0	0	0	0	0.12985	0	0	0	0	0	0	0	0	0	0	0	0.099973	0	0	0	0	0	0	0	0	0
k__Bacteria;p__Bacteroidetes;c__Flavobacteriia;o__Flavobacteriales;f__Flavobacteriaceae;g__Flavobacterium	*Unclassified Flavobacterium*	0	0	0	0	0	0	0	0	0	0	0	0	0	0	0	0	0	0	0	0	0	0	0	0	0	0.151003	0	0	0	0	0	0	0	0
k__Bacteria;p__Actinobacteria;c__Actinobacteria;o__Actinomycetales;f__Microbacteriaceae;g__Microbacterium;__	*Unclassified Microbacterium*	0	0	0	0	0	0	0	0	0	0	0	0	0	0	0	0	0	0	0	0	0	0	0	0	0	0.139566	0	0	0	0	0	0	0	0
k__Bacteria;p__Proteobacteria;c__Alphaproteobacteria;o__Sphingomonadales;f__Sphingomonadaceae;g__Sphingomonas	*Unclassified Sphingomonas*	0	0	0	0	0	0	0	0	0	0	0	0	0	0	0	0	0	0	0	0	0	0	0	0.089903	0	0.110961	0	0	0.005267	0	0	0	0	0
k__Bacteria;p__Firmicutes;c__Bacilli;o__Lactobacillales;f__Streptococcaceae;g__Streptococcus;__	*Unclassified Streptococcus*	0	0	0.018415	0	0.018495	0	0	0	0	0	0	0.05248	0.015166	0	0	0	0	0	0	0	0.076774	0	0	0	0	0	0	0	0	0	0	0	0.058418	0.046759
k__Bacteria;p__Bacteroidetes;c__Flavobacteriia;o__Flavobacteriales;f__[Weeksellaceae];g__Cloacibacterium	*Unclassified Cloacibacterium*	0	0	0	0	0	0	0	0	0	0	0	0	0	0	0	0	0.113728	0	0	0	0	0	0	0	0	0	0	0	0	0	0	0	0	0
k__Bacteria;p__Proteobacteria;c__Betaproteobacteria;o__Neisseriales;f__Neisseriaceae;g__Neisseria;s__subflava	*Neisseria subflava*	0	0	0	0	0	0	0	0	0.009157	0	0	0	0	0	0	0	0	0	0	0.197944	0	0	0	0	0.00577	0	0	0	0	0	0	0	0	0
k__Bacteria;p__Firmicutes;c__Bacilli;o__Lactobacillales;f__Lactobacillaceae;g__Lactobacillus;s__iners	*Lactobacillus iners*	2.45E-05	0	0	0	0	0	2.1E-05	0.234493	0	0	0	0	0	0	0	0	0	0	0	0	0	0	2.87E-05	0	0	0	0	0	0.010139	0	0	0	0	0
k__Bacteria;p__Proteobacteria;c__Alphaproteobacteria;o__Caulobacterales;f__Caulobacteraceae;g__Brevundimonas;s__vesiculari	*Brevundimonas vesicularis*	0	0	0	0	0	0	0	0	0	0	0	0	0	0	0	0	0	0	0	0	0	0	0	0	0	0.101189	0	0	0	0	0	0	0	0
k__Bacteria;p__Bacteroidetes;c__Cytophagia;o__Cytophagales;f__Cytophagaceae;g__Hymenobacter	*Unclassified Hymenobacter*	0	0	0	0	0	0	0.06906	0	0	0	0	0	0	0	0	0	0	0	0	0	0	0	0	0	0	0	0	0	0	0	0	0	0	0
k__Bacteria;p__Proteobacteria;c__Betaproteobacteria;o__Burkholderiales;f__Comamonadaceae;g__Variovorax;s__paradoxu	*Variovorax paradoxus*	0	0	0	0	0	0	0	0	0	0	0	0	0	0	0	0	0	0	0	0	0	0	0	0	0	0.096391	0	0	0	0	0	0	0	0
k__Bacteria;p__Firmicutes;c__Bacilli;o__Bacillales;f__Bacillaceae;g__Anoxybacillus;s__kestanbolensis	*Anoxybacillus kestanbolensis*	0	0	0	0	0	0	0	0	0	0	0	0	0	0	0	0	0	0	0	0	0	0	0	0	0	0	0	0	0	0.095746	0	0	0	0
k__Bacteria;p__Proteobacteria;c__Alphaproteobacteria;o__Rhizobiales;f__Beijerinckiaceae;;__	*Unclassified Beijerinckiaceae*	0	0	0	0	0	0	0	0	0.163443	0	0	0	0	0	0	0	0	0	0	0	0	0	0	0	0	0	0	0	0	0	0	0	0	0
k__Bacteria;p__Actinobacteria;c__Actinobacteria;o__Actinomycetales;f__Frankiaceae;;__	*Unclassified Frankiaceae*	0	0	0	0	0	0	0	0	0	0	0	0	0	0	0	0	0	0	0.100439	0	0	0	0	0	0	0	0	0	0	0	0	0	0	0
k__Bacteria;p__Firmicutes;c__Bacilli;o__Lactobacillales;f__Streptococcaceae;g__Streptococcus	*Unclassified Streptococcus*	0	0	0.083842	0	0.0821	0	0	0	0	0	0.119114	0	0	0	0	0	0	0	0	0	0	0.034506	0	0	0	0	0	0	0	0	0	0	0.002988	0.075874
k__Bacteria;p__Actinobacteria;c__Actinobacteria;o__Actinomycetales;f__Propionibacteriaceae;;__	*Unclassified Propionibacteriaceae*	0	0	0	0	0	0	0	0	0	0	0	0	0	0	0	0	0	0	0	0	0	0	0	0	0.068399	0	0	0	0	0	0	0	0	0
k__Bacteria;p__Actinobacteria;c__Actinobacteria;o__Actinomycetales;f__Actinomycetaceae;g__N09	*Unclassified N09*	0	0	0	0	0	0	0	0	0	0	0	0	0	0	0	0	0	0	0	0	0	0	0	0	0.062208	0	0	0	0	0	0	0	0	0
k__Bacteria;p__Bacteroidetes;c__Sphingobacteriia;o__Sphingobacteriales;f__Sphingobacteriaceae;;__	*Unclassified Sphingobacteriaceae*	0	0	0	0	0	0	0	0	0	0	0	0	0	0	0	0	0	0	0	0	0	0	0	0.049571	0	0	0	0	0	0	0	0	0	0
k__Bacteria;p__Proteobacteria;c__Alphaproteobacteria;o__Sphingomonadales;f__Sphingomonadaceae;g__Sphingomonas;s__wittich	*Sphingomonas wittichii*	0	0	0	0	0	0	0	0	0	0	0	0	0	0	0	0	0	0	0	0	0	0	0	0	0	0.057076	0	0	0	0	0	0	0	0
k__Bacteria;p__Firmicutes;c__Bacilli;o__Lactobacillales;f__Enterococcaceae;g__Enterococcus;s__cecorum	*Enterococcus cecorum*	0	0	0	0	0	0	0	0	0	0	0.181124	0	0	0	0	0	0	0	0	0	0	0	0	0	0	0	0	0	0	0	0	0	0	0
k__Bacteria;p__Proteobacteria;c__Alphaproteobacteria;o__Rhizobiales;f__Methylobacteriaceae;;__	*Unclassified Methylobacteriaceae*	0	0	0	0	0	0	0	0	0	0	0	0	0	0	0	0	0	0	0	0	0	0	0	0	0	0.052965	0	0	0	0	0	0	0	0
k__Bacteria;p__Proteobacteria;c__Alphaproteobacteria;o__Rhizobiales;f__Bradyrhizobiaceae;g__Bradyrhizobium	*Unclassified Bradyrhizobium*	0	0	0	0	0	0	0	0	0	0	0	0	0	0	0	0	0	0	0.057568	0	0	0	0	0	0	0	0	0	0	0	0	0	0	0
k__Bacteria;p__Actinobacteria;c__Actinobacteria;o__Actinomycetales;f__Actinomycetaceae;g__Actinomyces	*Unclassified Actinomyces*	0	0	0.002238	0	0.000482	0	0	0	0	0	0	0	0	0	0	0	0	0	0	0.088102	0	0	0	0	0	0	0	0	0.017422	0	0	0	0	0
k__Bacteria;p__Proteobacteria;c__Alphaproteobacteria;o__Rhizobiales;f__Methylobacteriaceae;g__Methylobacterium;s__adhaesivum	*Methylobacterium adhaesivum*	0	0	0	0	0	0	0	0	0	0	0	0	0	0	0	0	0	0	0	0	0	0	0	0	0	0.035372	0	0	0	0	0	0	0	0
k__Bacteria;p__Firmicutes;c__Bacilli;o__Lactobacillales;f__Streptococcaceae;g__Streptococcus;s__infantis	*Streptococcus infantis*	0	0	0	0	0.065738	0	0	0	0	0	0	0	0	0	0	0	0	0	0	0	0	0	0	0	0	0	0	0	0	0	0	0	0	0
k__Bacteria;p__Actinobacteria;c__Actinobacteria;o__Actinomycetales;f__Corynebacteriaceae;g__Corynebacterium	*Unclassified Corynebacterium*	0	0.005971	0	0.118879	0	0	0	0	0	0	0	0	0	0	0	0	0	0	0	0	0	0	0	0	0	0	0	0	0	0	0	0	0	0
k__Bacteria;p__Proteobacteria;c__Betaproteobacteria;o__Burkholderiales;f__Alcaligenaceae;g__Pigmentiphaga	*Unclassified Pigmentiphaga*	0	0	0	0	0	0	0	0	0	0	0	0	0	0	0	0	0	0	0	0	0	0	0	0	0	0.026969	0	0	0	0	0	0	0	0
k__Bacteria;p__Proteobacteria;c__Gammaproteobacteria;o__Pasteurellales;f__Pasteurellaceae;g__Haemophilus;s__parainfluenza	*Haemophilus parainfluenzae*	0	0	0.007283	0	0.087619	0	0	0	0	0	0.00525	0	0	0	0	0	0	0	0	0	0	0	0	0	0	0	0	0	0	0	0	0	0	0
k__Bacteria;p__Actinobacteria;c__Actinobacteria;o__Actinomycetales;f__Micrococcaceae;g__Micrococcus	*Unclassified Micrococcus*	0	0	0	0	0	0	0	0	0	0	0	0	0.019059	0	0	0	0	0	0	0	0	0	0	0	0	0	0	0	0	0	0	0	0	0
k__Bacteria;p__Proteobacteria;c__Betaproteobacteria;o__Neisseriales;f__Neisseriaceae;g__Neisseria;__	*Unclassified Neisseria*	0	0	0	0	0	0	0	0	0	0	0	0	0	0	0	0	0	0	0	0.05127	0	0	0	0	0	0	0	0	0	0	0	0.001572	0	0
k__Bacteria;p__Firmicutes;c__Bacilli;o__Bacillales;f__Paenibacillaceae;g__Paenibacillus	*Unclassified Paenibacillus*	0	0	0	0	0	0	0	0	0	0	0	0	0	0	0	0	0	0	0	0	0	0	0	0	0	0.021663	0	0	0	0	0	0	0	0
k__Bacteria;p__Proteobacteria;c__Gammaproteobacteria;o__Pseudomonadales;f__Moraxellaceae;g__Enhydrobacter	*Unclassified Enhydrobacter*	0	0	0	0	0	0	0	0	0	0	0	0	0	0	0	0	0	0	0	0	0	0	0	0	0	0	0	0	0	0	0	0	0	0.047117
k__Bacteria;p__Firmicutes;c__Bacilli;o__Bacillales;f__[Exiguobacteraceae];g__Exiguobacterium	*Unclassified Exiguobacterium*	0	0	0	0	0	0	0	0.025534	0	0	0	0	0	0	0	0	0	0	0	0	0	0	0	0	0	0	0	0	0	0	0	0	0	0
k__Bacteria;p__Firmicutes;c__Bacilli;o__Gemellales;f__Gemellaceae;__;__	*Unclassified Gemellaceae*	0	0	0	0	0.046219	0	0	0	0	0	0	0	0	0	0	0	0	0	0	0	0	0	0	0	0	0	0	0	0	0	0	0	0	0
k__Bacteria;p__Actinobacteria;c__Actinobacteria;o__Actinomycetales;f__Micrococcaceae;g__Micrococcus;__	*Unclassified Micrococcus*	0	0	0	0	0	0	0	0	0	0	0	0	0.012177	0	0	0	0	0	0	0	0	0	0	0	0	0	0	0	0	0	0	0	0	0
k__Bacteria;p__Proteobacteria;c__Gammaproteobacteria;o__Enterobacteriales;f__Enterobacteriaceae;g__Enterobacter;__	*Unclassified Enterobacter*	0	0	0	0	0	0	0	0	0	0	0	0	0	0	0	0.035453	0	0	0	0	0	0	0	0	0	0	0	0	0	0	0	0	0	0
k__Bacteria;p__Actinobacteria;c__Actinobacteria;o__Actinomycetales;f__Micrococcaceae;g__Nesterenkonia	*Unclassified Nesterenkonia*	0	0	0	0	0	0	0	0	0	0	0	0	0	0	0	0.034106	0	0	0	0	0	0	0	0	0	0	0	0	0	0	0	0	0	0
k__Bacteria;p__Proteobacteria;c__Gammaproteobacteria;o__Pseudomonadales;f__Pseudomonadaceae;g__Pseudomonas	*Unclassified Pseudomonas*	0	0	0	0	0	0	0	0	0	0	0	0	0.009572	0	0	0	0	0	0	0	0	0	0	0	0	0	0	0	0	0	0	0	0	0
k__Bacteria;p__[Thermi];c__Deinococci;o__Thermales;f__Thermaceae;g__Meiothermus	*Unclassified Meiothermus*	0	0	0	0	0	0	0	0	0	0	0	0	0	0	0	0	0	0	0	0	0	0	0	0	0	0	0	0	0	0	0	0	0.032793	0
k__Bacteria;p__Proteobacteria;c__Alphaproteobacteria;o__Rhodospirillales;f__Acetobacteraceae;g__Roseomonas	*Unclassified Roseomonas*	0	0	0	0	0	0	0	0	0	0	0	0	0	0	0	0	0	0	0	0	0	0	0	0	0	0.012462	0	0	0	0	0	0	0	0
k__Bacteria;p__Proteobacteria;c__Gammaproteobacteria;o__Pseudomonadales;f__Moraxellaceae;g__Acinetobacter	*Unclassified Acinetobacter*	0	0	0	0	0	0	0	0	0	0	0	0	0	0	0	0	0	0	0	0	0	0	0	0	0	0	0	0	0	0	0	0.025891	0	0
k__Bacteria;p__Proteobacteria;c__Gammaproteobacteria;o__Enterobacteriales;f__Enterobacteriaceae;g__Klebsiella;s__oxytoc	*Klebsiella oxytoca*	0	0	0	0	0	0	0	0	0	0	0	0	0	0	0	0	0	0	0	0	0.021943	0	0	0	0	0	0	0	0	0	0	0	0	0
k__Bacteria;p__Proteobacteria;c__Alphaproteobacteria;o__Sphingomonadales;f__Sphingomonadaceae;g__Novosphingobium	*Unclassified Novosphingobium*	0	0	0	0	0	0	0	0	0	0	0	0	0	0	0	0	0	0	0	0	0	0	0	0	0	0.0116	0	0	0	0	0	0	0	0
k__Bacteria;p__Bacteroidetes;c__Flavobacteriia;o__Flavobacteriales;f__[Weeksellaceae];g__Chryseobacterium	*Unclassified Chryseobacterium*	0	0	0.040232	0	0	0	0	0	0	0	0	0	0	0	0	0	0	0	0	0	0	0	0	0	0	0	0	0	0	0	0	0	0	0
k__Bacteria;p__Firmicutes;c__Bacilli;o__Bacillales;f__Paenibacillaceae;g__Brevibacillus;__	*Unclassified Brevibacillus*	0	0	0	0	0	0	0	0	0	0	0	0	0	0	0	0	0	0	0	0	0	0	0	0	0	0.021489	0	0	0	0	0	0	0	0
k__Bacteria;p__Proteobacteria;c__Gammaproteobacteria;o__Enterobacteriales;f__Enterobacteriaceae;__;__	*Unclassified Enterobacteriaceae*	0	0	0.010103	0	0	0	0	0	0	0	0	0	0	0	0	0	0	0	0	0	0.021628	0	0	0	0	0	0	0	0	0	0	0	0	0
k__Bacteria;p__Actinobacteria;c__Acidimicrobiia;o__Acidimicrobiales;f__C111;;__	*Unclassified C111*	0	0	0	0	0	0	0	0	0	0	0	0	0.006433	0	0	0	0	0	0	0	0	0	0	0	0	0	0	0	0	0	0	0	0	0
k__Bacteria;p__Proteobacteria;c__Betaproteobacteria;o__Burkholderiales;f__Comamonadaceae;g__Delftia	*Unclassified Delftia*	0	0	0	0	0	0	0	0	0	0	0	0	0	0	0	0.018854	0	0	0	0	0	0	0	0	0	0	0	0	0	0	0	0	0	0
k__Bacteria;p__Firmicutes;c__Bacilli;o__Bacillales;f__Paenibacillaceae;g__Brevibacillus	*Unclassified Brevibacillus*	0	0	0	0	0	0	0	0	0	0	0	0	0	0	0	0	0	0	0	0	0	0	0	0	0	0.008432	0	0	0	0	0	0	0	0
k__Bacteria;p__Firmicutes;c__Bacilli;o__Lactobacillales;f__Lactobacillaceae;g__Lactobacillus;s__helveticus	*Lactobacillus;s__helveticus*	0	5.19E-05	0	0	0	3.38E-05	0	0	0	0	0.019425	0	0	0	0	0	0	0	0	0	0	0	0	0	0	0	0	0	0	0	0	0	0	0
k__Bacteria;p__Proteobacteria;c__Betaproteobacteria;o__Burkholderiales;f__Comamonadaceae;g__Pelomonas	*Unclassified Pelomonas*	0	0	0	0	0	0.002414	0	0	0	0	0	0	0	0	0	0	0	0	0	0	0	0	0	0	0	0	0	0	0	0	0	0	0	0
k__Bacteria;p__Proteobacteria;c__Alphaproteobacteria;o__Sphingomonadales;f__Sphingomonadaceae;g__Sphingomonas;_	*Unclassified Sphingomonas*	0	0	0	0	0	0	0	0	0	0	0	0	0	0	0	0	0	0	0	0	0	0	0	0	0	0.002184	0	0	0	0	0	0	0	0
k__Bacteria;p__Firmicutes;c__Bacilli;o__Bacillales;f__Bacillaceae;g__Bacillus;__	*Unclassified Bacillus*	0	0	0	0	0	0	0	0	0	0	0	0	0.000143	0	0.000193	0.000272	0.000285	0.000314	0.000261	0	0	0	0	0	0	0	0	0.00126	0	0	0.000159	0	0	0
k__Bacteria;__;__;__;__;__	*Unclassified Bacteria*	0	0	0	0	0	0	0	0	0	0	0	0	0	0	0	0	0.000564	0.002366	0	0	0	0	0	0	0	0	0	0	0	0	0	0	0	0
k__Bacteria;p__Firmicutes;c__Bacilli;o__Lactobacillales;f__Lactobacillaceae;g__Lactobacillus;s__plantarum	*Lactobacillus plantarum*	0	0	0	0	0	0	0	0	0	0	0	0	0	0	0	0	0	0	0	0	0.001725	0.002103	0	0	0	0	0	0	0	0	0	0	0	0
k__Bacteria;p__Firmicutes;c__Bacilli;o__Bacillales;f__Thermoactinomycetaceae;;__	*Unclassified Thermoactinomycetaceae*	0	0	0	0	0	0	0	0	0	0.001366	0	0	0	0	0	0	0	0	0	0	0	0	0	0	0	0	0	0	0	0	0	0	0	0
k__Bacteria;p__Firmicutes;c__Bacilli;o__Gemellales;f__Gemellaceae;;__	*Unclassified Gemellaceae*	0	0	0	0	0.001807	0	0	0	0	0	0	0	0	0	0	0	0	0	0	0	0	0	0	0	0	0	0	0	0	0	0	0	0	0
k__Bacteria;p__Proteobacteria;c__Alphaproteobacteria;o__Rhizobiales;f__Methylobacteriaceae;g__Methylobacterium;_	*Unclassified Methylobacterium*	0	0	0	0	0	0	0	0	0	0	0	0	0	0	0	0	0	0	0	0	0	0	0	0	0	0.000786	0	0	0	0	0	0	0	0
k__Bacteria;p__Firmicutes;c__Bacilli;o__Bacillales;f__Paenibacillaceae;g__Paenibacillus;__	*Unclassified Paenibacillus*	0	0	0	0	0	0	0	0	0	0	0	0	0	0	0	0	0	0	0	0	0	0	0	0	0	0.000722	0	0	0	0	0	0	0	0
k__Bacteria;p__Firmicutes;c__Bacilli;o__Bacillales;f__[Exiguobacteraceae];__;__	*Unclassified [Exiguobacteraceae]*	0	0	0	0	0	0	0	0.000954	0	0	0	0	0	0	0	0	0	0	0	0	0	0	0	0	0	0	0	0	0	0	0	0	0	0

Table S1: Taxonomic identification with relative abundance data.
